# Supplementary material for: EB1 decoration of microtubule lattice facilitates spindle-kinetochore lateral attachment in Plasmodium male gametogenesis
Source: Nat Commun. 2023 May 19;14:2864. doi: 10.1038/s41467-023-38516-3 (PMC10199041; doi:10.1038/s41467-023-38516-3)
Supplement: Supplementary file 1 — Supplementary Information [file 41467_2023_38516_MOESM1_ESM.pdf]

Supplemental Information

EB1 decoration of microtubule lattice facilitates spindle-kinetochore lateral attachment in Plasmodium male gametogenesis

Shuzhen Yang[1, 4], Mengya Cai[1, 4], Junjie Huang[2, 4], Shengnan Zhang[1], Xiaoli Mo[1], Kai Jiang[2, 3,*], Huiting Cui [1,*], Jing Yuan[1,*]

1. Supplementary Figures 1-13 and figure legends
2. Supplementary Table 1 Primers and oligonucleotides used in this study
3. Supplementary Table 2 List of genetically modified parasite strains used in this study

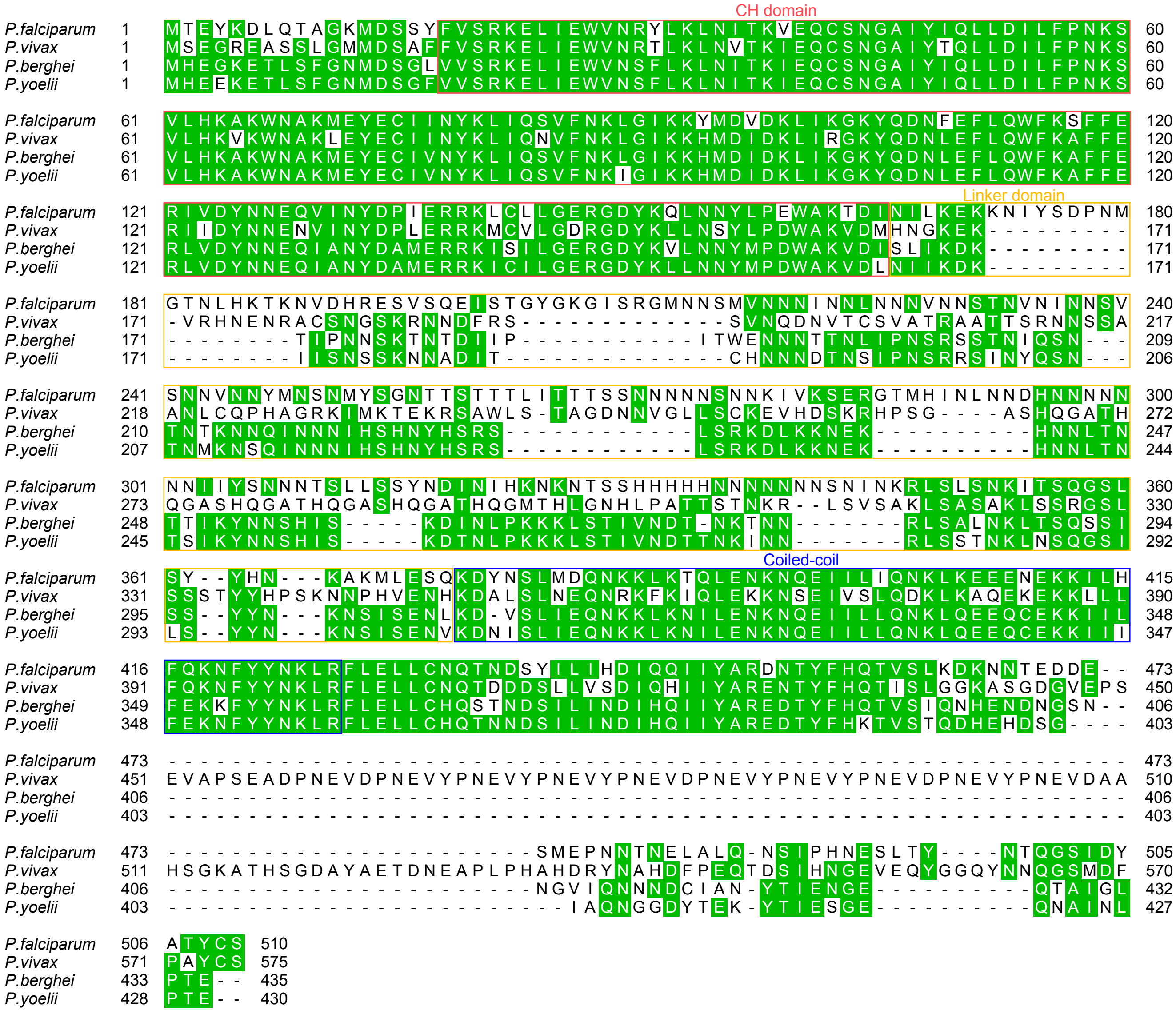
CH domain
Linker domain
Coiled-coil
P.falciparum 1 MTEYKDLQTAGKMDSSYFVSRKELIEWVNRYLKLNITKVEQCSNGAIYIQLLDILFPNKS 60
P.vivax 1 MSEGREASSLGMMDSAFFVSRKELIEWVNRTLKLNVTKIEQCSNGAIYTQLLDILFPNKS 60
P.berghei 1 MHEGKETLSFGNMDSGLVVSRKELIEWVNSFLKLNITKIEQCSNGAIYIQLLDILFPNKS 60
P.yoelii 1 MHEEKETLSFGNMDSGFVVSRKELIEWVNSFLKLNITKIEQCSNGAIYIQLLDILFPNKS 60
P.falciparum 61 VLHKAKWNAKMEYECIINYKLIQSVFNKLGIKKYMDVDKLIKGKYQDNFEFLQWFKSFFE 120
P.vivax 61 VLHKVKWNAKLEYECIINYKLIQNVFNKLGIKKHMDIDKLIRGKYQDNLEFLQWFKAFFE 120
P.berghei 61 VLHKAKWNAKMEYECIVNYKLIQSVFNKLGIKKHMDIDKLIKGKYQDNLEFLQWFKAFFE 120
P.yoelii 61 VLHKAKWNAKMEYECIVNYKLIQSVFNKIGIKKHMDIDKLIKGKYQDNLEFLQWFKAFFE 120
P.falciparum 121 RIVDYNNEQVINYDPIERRKLCLLGERGDYKQLNNYLPEWAKTDINILKEKKNIYSDPNM 180
P.vivax 121 RIIDYNNENVINYDPLERRKMCVLGDRGDYKLLNSYLPDWAKVDMHNGKEK--------- 171
P.berghei 121 RLVDYNNEQIANYDAMERRKISILGERGDYKVLNNYMPDWAKVDISLIKDK--------- 171
P.yoelii 121 RLVDYNNEQIANYDAMERRKICILGERGDYKLLNNYMPDWAKVDLNIIKDK--------- 171
P.falciparum 181 GTNLHKTKNVDHRESVSQEISTGYGKGISRGMNNSMVNNNINNLNNNVNNSTNVNINNSV 240
P.vivax 171 -VRHNENRACSNGSKRNNDFRS-------------SVNQDNVTCSVATRAATTSRNNSSA 217
P.berghei 171 ---------TIPNNSKTNTDIIP----------ITWENNNTTNLIPNSRSSTNIQSN--- 209
P.yoelii 171 ---------IISNSSKNNADIT-------------CHNNNDTNSIPNSRRSINYQSN--- 206
P.falciparum 241 SNNVNNYMNSNMYSGNTTSTTTLITTTSSNNNNNSNNKIVKSERGTMHINLNNDHNNNNN 300
P.vivax 218 ANLCQPHAGRKIMKTEKRSAWLS-TAGDNNVGLLSCKEVHDSKRHPSG----ASHQGATH 272
P.berghei 210 TNTKNNQINNNIHSHNYHSRS-----------LSRKDLKKNEK-----------HNNLTN 247
P.yoelii 207 TNMKNSQINNNIHSHNYHSRS-----------LSRKDLKKNEK-----------HNNLTN 244
P.falciparum 301 NNIIYSNNNTSLLSSYNDINIHKNKNTSSHHHHHNNNNNNNSNINKRLSLSNKITSQGSL 360
P.vivax 273 QGASHQGATHQGASHQGATHQGMTHLGNHLPATTSTNKR--LSVSAKLSASAKLSSRGSL 330
P.berghei 248 TTIKYNNSHIS-----KDINLPKKKLSTIVNDT-NKTNN-------RLSALNKLTSQSSI 294
P.yoelii 245 TSIKYNNSHIS-----KDTNLPKKKLSTIVNDTTNKINN-------RLSSTNKLNSQGSI 292
P.falciparum 361 SY--YHN---KAKMLESQKDYNSLMDQNKKLKTQLENKNQEIILIQNKLKEEENEKKILH 415
P.vivax 331 SSSTYYHPSKNNPHVENHKDALSLNEQNRKFKIQLEKKNSEIVSLQDKLKAQEKEKKLLL 390
P.berghei 295 SS--YYN---KNSISENLKD-VSLIEQNKKLKNILENKNQEIILLQNKLQEEQCEKKIIL 348
P.yoelii 293 LS--YYN---KNSISENVKDNISLIEQNKKLKNILENKNQEIILLQNKLQEEQCEKKIII 347
P.falciparum 416 FQKNFYYNKLRFLELLCNQTNDSYILIHDIQQIIYARDNTYFHQTVSLKDKNNTEDDE-- 473
P.vivax 391 FQKNFYYNKLRFLELLCNQTDDDSLLVSDIQHIIYARENTYFHQTISLGGKASGDGVEPS 450
P.berghei 349 FEKKFYYNKLRFLELLCHQSTNDSILINDIHQIIYAREDTYFHQTVSIQNHENDNGSN-- 406
P.yoelii 348 FEKNFYYNKLRFLELLCHQTNNDSILINDIHQIIYAREDTYFHKTVSTQDHEHDSG---- 403
P.falciparum 473 -- 473
P.vivax 451 EVAPSEADPNEVDPNEVYPNEVYPNEVYPNEVDPNEVYPNEVYPNEVDPNEVYPNEVDAA 510
P.berghei 406 -- 406
P.yoelii 403 -- 403
P.falciparum 473 -----------------------SMEPNNTNELALQ-NSIPHNESLTY----NTQGSIDY 505
P.vivax 511 HSGKATHSGDAYAETDNEAPLPHAHDRYNAHDFPEQTDSIHNGEVEQYGGQYNNQGSMDF 570
P.berghei 406 -----------------------NGVIQNNNDCIAN-YTIENGE----------QTAIGL 432
P.yoelii 403 -------------------------IAQNGGDYTEK-YTIESGE----------QNAINL 427
P.falciparum 506 ATYCS 510
P.vivax 571 PAYCS 575
P.berghei 433 PTE-- 435
P.yoelii 428 PTE-- 430

Supplementary figure legend

Supplementary Figure 1. EB1 amino acid sequence alignment among four *Plasmodium* parasites.

EB1 protein sequences for alignment are from four *Plasmodium* species: the *P. falciparum* (PF3D7_0307300), *P. vivax* (PVX_119485), *P. berghei* (PBANKA_ 0405600), and *P. yoelii* (PY17X_0407900). The calponin homology (CH) domain (red box), the linker region (yellow box), and the coiled-coil (CC) domain (blue box) were indicated.

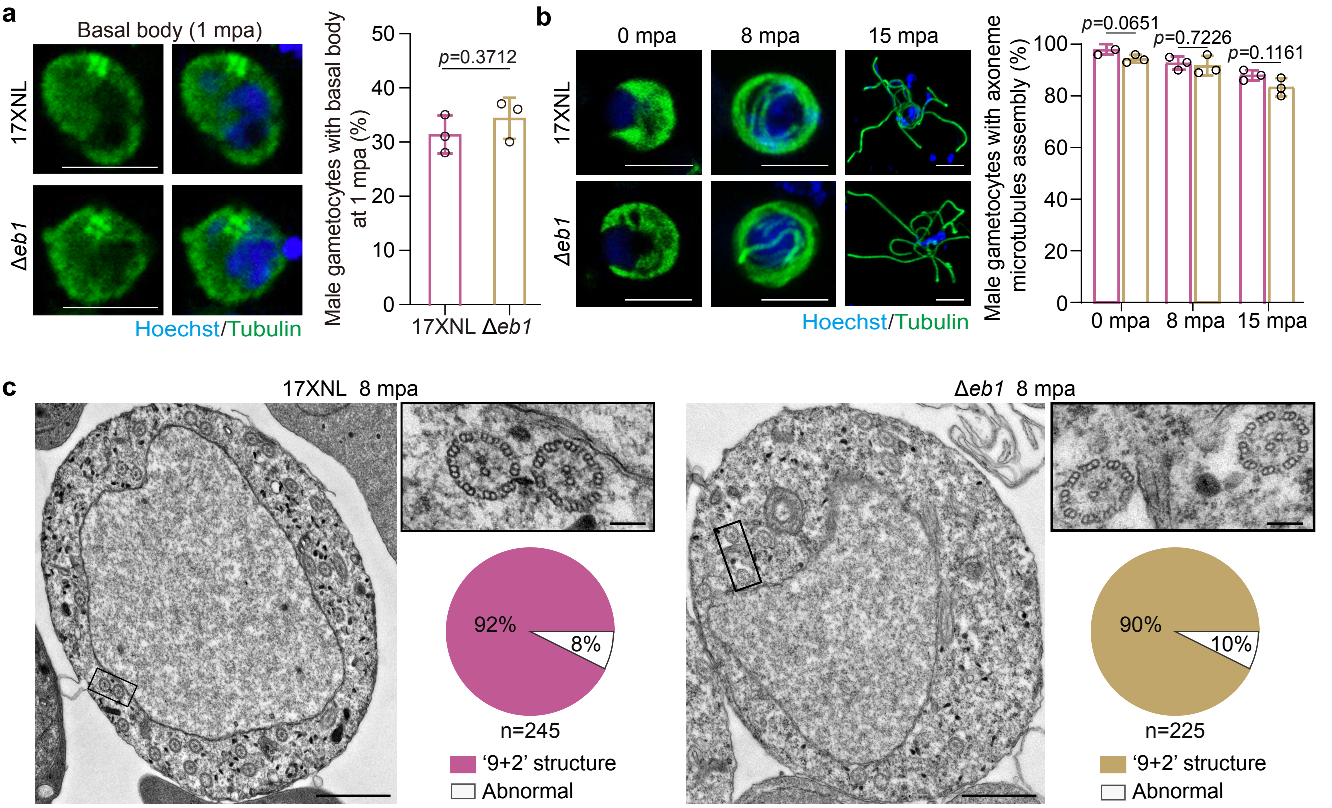
a
Basal body (1 mpa)
17XNL
Δeb1
Hoechst/Tubulin
Male gametocytes with basal body at 1 mpa (%)
p=0.3712
17XNL
Δeb1
b
0 mpa
8 mpa
15 mpa
17XNL
Δeb1
Hoechst/Tubulin
Male gametocytes with axoneme microtubules assembly (%)
p=0.0651
p=0.7226
p=0.1161
0 mpa
8 mpa
15 mpa
c
17XNL 8 mpa
Δeb1 8 mpa
92%
8%
n=245
'9+2' structure
Abnormal
90%
10%
n=225
'9+2' structure
Abnormal

Supplementary Figure 2. EB1-null parasites develop cytosolic axoneme during male gametogenesis

a Basal body formation in male gametocytes at 1 mpa. Parasites were co-stained with Hoechst 33342 and antibody against both α-Tubulin I and α-Tubulin II (male gametocyte marker). A typical basal body tetramer was observed close to the nucleus. Scale bar = 5 μm. Right panel indicates the percentage of male gametocytes showing the tetramer. Mean ± SD from three independent experiments, two-tailed t-test.

b Cytosolic axoneme formation in male gametocytes at 0, 8, and 15 mpa. Parasites were co-stained with Hoechst 33342 and antibody against both α-Tubulin I and α-Tubulin II (male gametocyte marker). Scale bar = 5 μm. Right panel indicates the percentage of male gametocytes with cytosolic axoneme assembled. Mean ± SD from three independent experiments, two-tailed t-test.

c Transmission electron microscopy detecting the cytosolic axoneme in *17XNL* and Δ*eb1* male gametocytes at 8 mpa. Scale bar = 1 μm. Insets show enlargements of the boxed areas displaying the '9+2' MT structure of axonemes. Scale bar = 100 nm. Pie charts show the percentage of axonemes with '9+2' structure in each parasite. n is the number of axonemes counted.

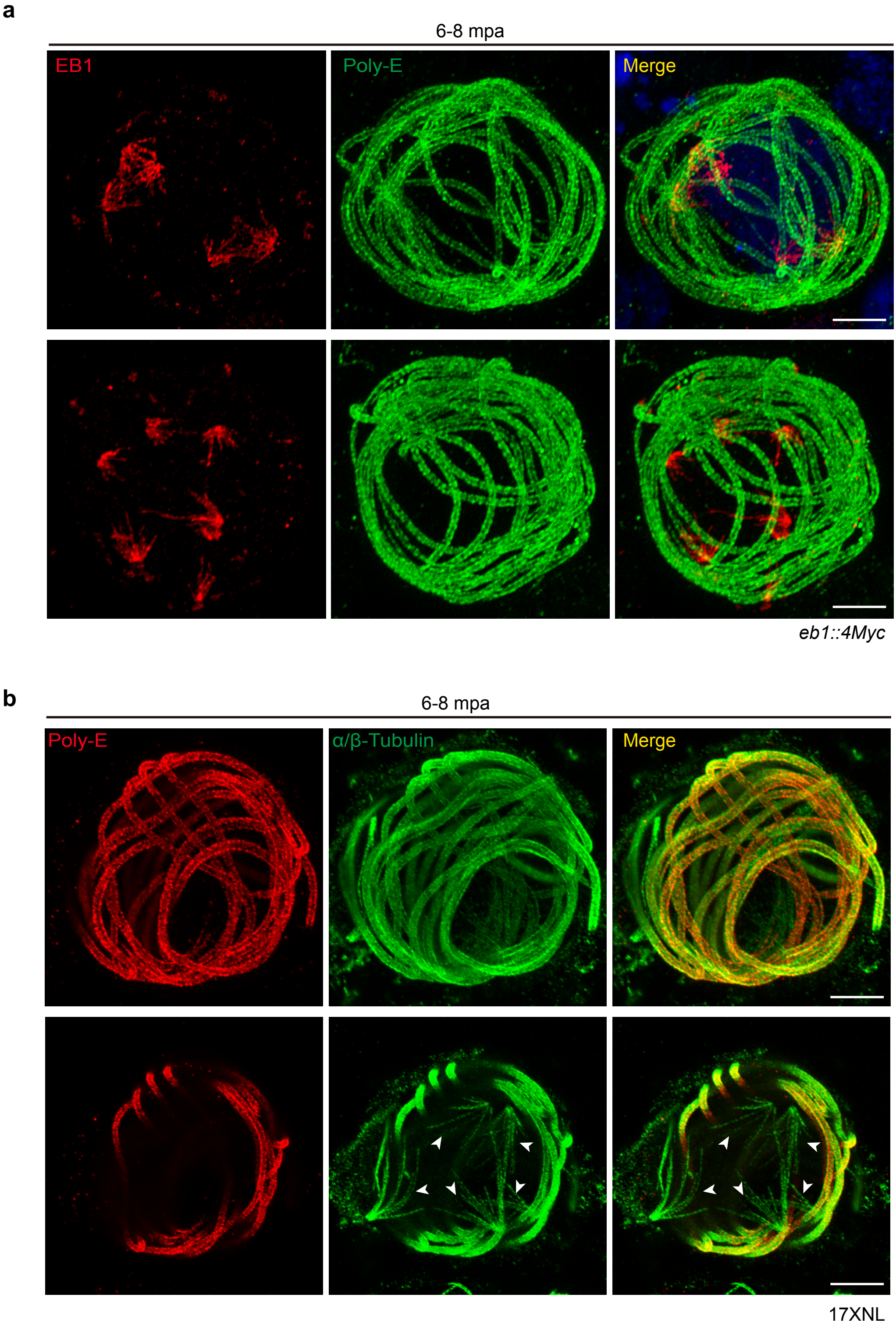
a
6-8 mpa
EB1
Poly-E
Merge
eb1::4Myc
b
6-8 mpa
Poly-E
α/β-Tubulin
Merge
17XNL

Supplementary Figure 3. U-ExM detection of spindle in activated male gametocytes

a U-ExM of hemispindle and axoneme in the *eb1::4Myc* male gametocytes at 6-8 mpa. Parasites were co-stained with antibodies against the Myc tag (red) and the polyglutamylated tubulin (PolyE, green). Hemispindles are EB1-positive and PolyE-negative while the axonemes are EB1-negative and PolyE-positive. Full section projections of fluorescent signal in parasites were collected. Representative maximum intensity projection (MIP) images were shown. Representative for three independent experiments. Scale bar = 5 μm.

b U-ExM of hemispindle and axoneme in the 17XNL male gametocytes at 6-8 mpa. Parasites were co-stained with antibodies against PolyE (red) and α/β-Tubulins (green). Hemspindles are tubulin-positive and PolyE-negative, enabling visualization of spindles in the activated male gametocytes. The upper panel shows the representative full projections of one male gametocytes, while the lower panel shows certain projections displaying the hemispindles (white arrows) in the same gametocyte. Representative for three independent experiments. Scale bar = 5 μm.

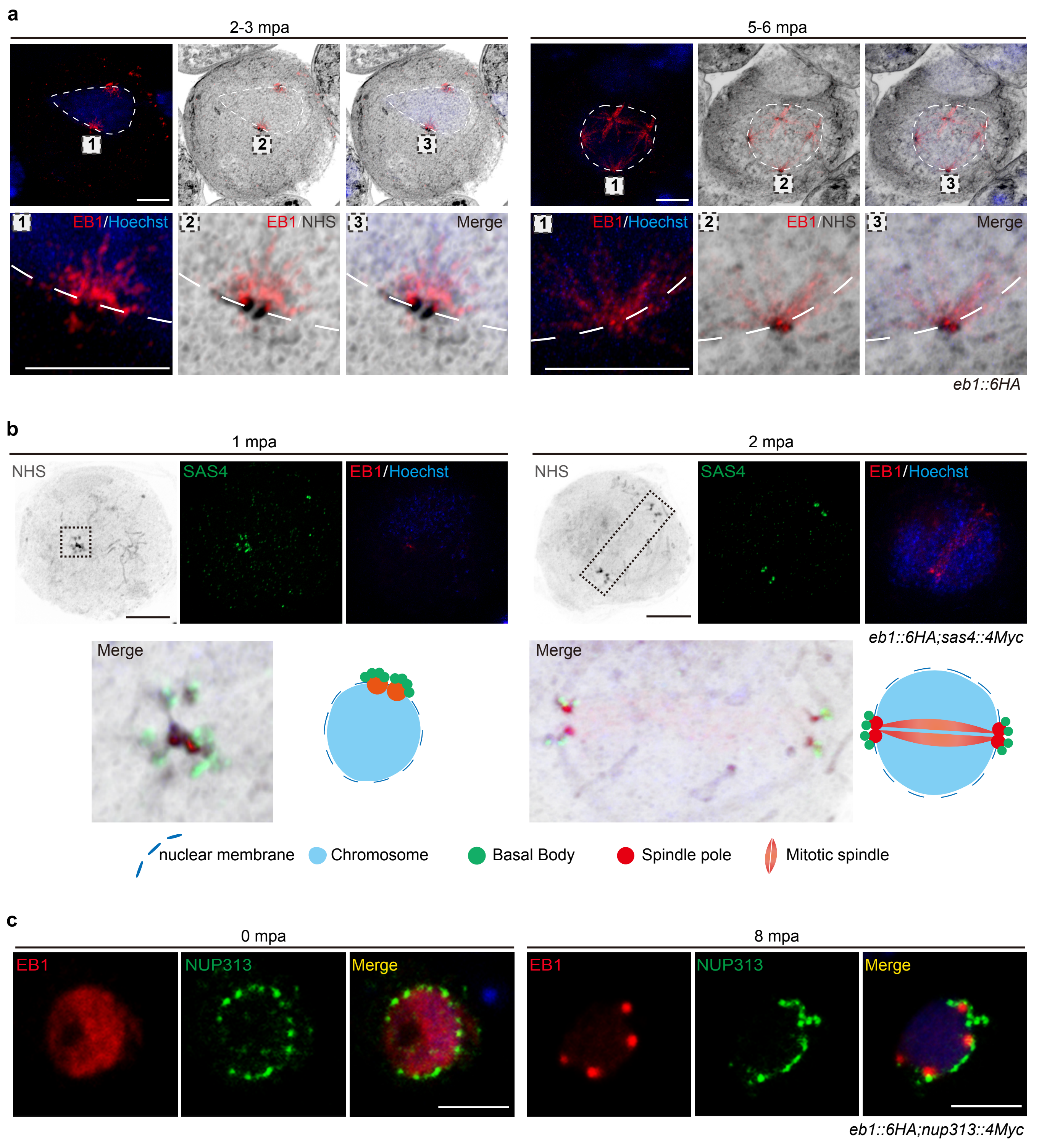
a
2-3 mpa
5-6 mpa
1
2
3
EB1/Hoechst
EB1/NHS
Merge
eb1::6HA
b
1 mpa
2 mpa
NHS
SAS4
EB1/Hoechst
eb1::6HA;sas4::4Myc
Merge
nuclear membrane
Chromosome
Basal Body
Spindle pole
Mitotic spindle
c
0 mpa
8 mpa
EB1
NUP313
Merge
eb1::6HA;nup313::4Myc

Supplementary Figure 4. Localization analysis of EB1-decorated spindle in activated male gametocytes

a U-ExM of EB1 expression in the *eb1::6HA* male gametocytes at 2-3 and 5-7 mpa. Parasites were co-stained with anti-HA antibody, Hoechst 33342, and protein NHS-ester dye. Nuclear area was indicated with a white dashed line. One NHS-ester signal dense region, representing a likely nuclear membrane embedded spindle pole, was zoomed in and shown in the low panels. Representative for three independent experiments. Scale bar = 5 μm.

b U-ExM of EB1 and SAS4 (basal body protein) in early stage activated male gametocytes of a double-tagged parasite strain *eb1::6HA*;*sas4::4Myc*. Parasites were co-stained with the antibodies against HA and Myc tags and Hoechst 33342. Insets show enlargements of the boxed areas. Representative for three independent experiments. Scale bar = 5 μm.

c IFA of EB1 and NUP313 (nuclear pore protein) in male gametocytes of a double-tagged parasite strain *eb1::6HA*;*nup313::4Myc*. Parasites were co-stained with antibodies against HA and Myc tags and Hoechst 33342. Representative for three independent experiments. Scale bar = 5 μm.

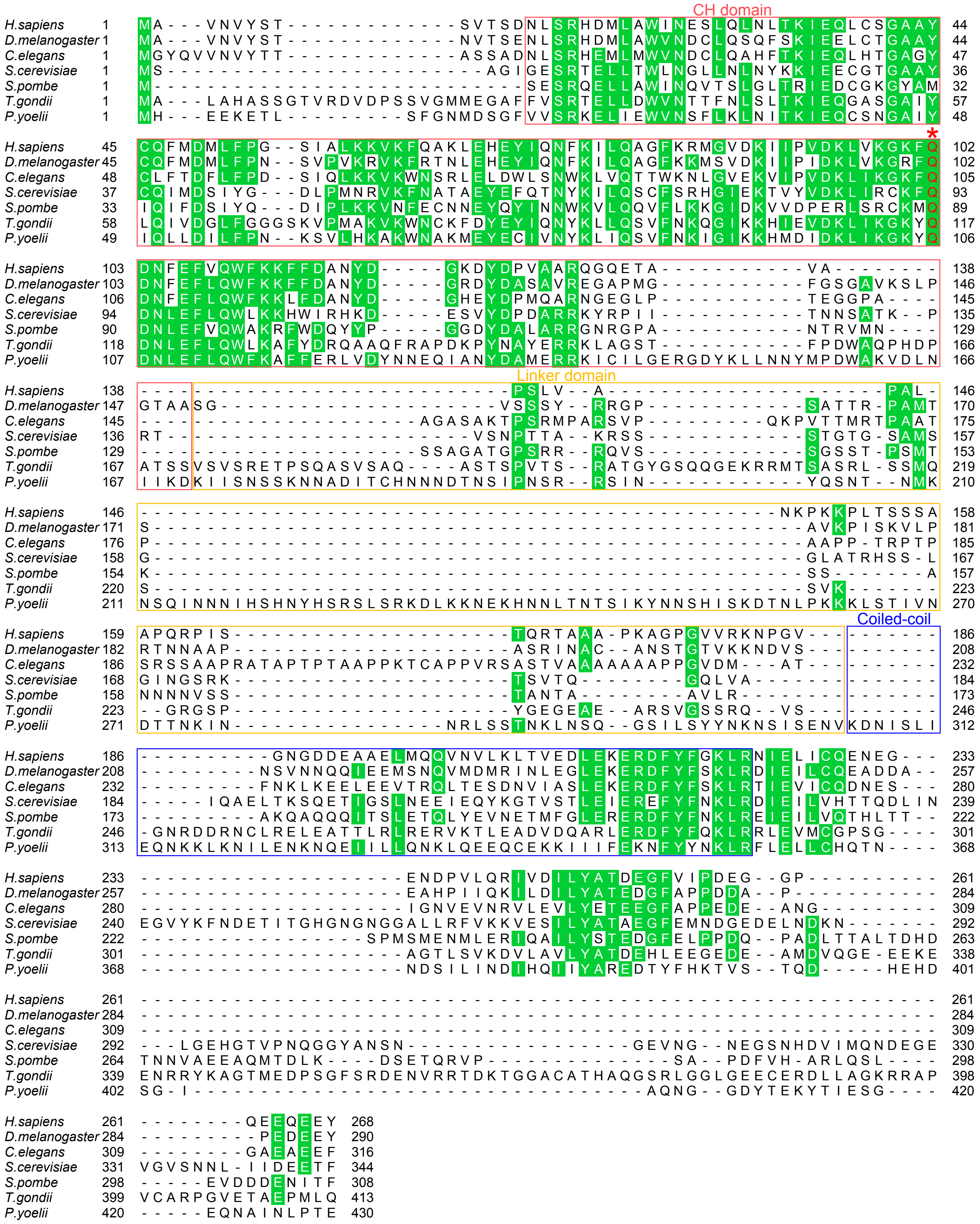
CH domain
Linker domain
Coiled-coil
H.sapiens
D.melanogaster
C.elegans
S.cerevisiae
S.pombe
T.gondii
P.yoelii

Supplementary Figure 5. Comparison of EB1 protein sequences between *Plasmodium* and eukaryotic model organisms

EB1 protein sequences for alignment are from *H. sapiens*, *D. melanogaster*, *C. elegans*, *S. cerevisiae,* *S. pombe, T.gondii* and *P. yoelii*. The calponin homology (CH) domain (red box), the linker region (yellow box), and the coiled-coil (CC) domain (blue box) were indicated. One residue glutamine (Q) at CH domain, which is conserved among EB1 proteins from all the eukaryotic organisms, was marked with a red star.

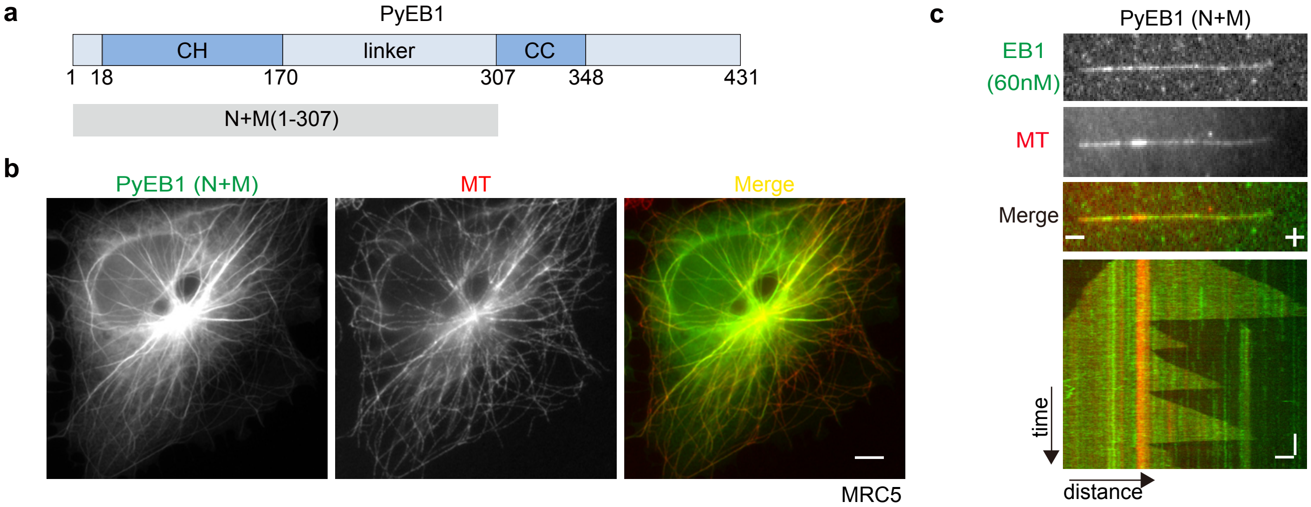

a
PyEB1
CH
linker
CC
1
18
170
307
348
431
N+M(1-307)
b
PyEB1 (N+M)
MT
Merge
MRC5
c
PyEB1 (N+M)
EB1
(60nM)
MT
Merge
time
distance

Supplementary Figure 6. Protein domain of PyEB1 critical for MT lattice affinity

a Protein domain organization of *P. yoelii* EB1 (PyEB1). The fragment of PyEB1-N+M (1-307 aa) was tested for MT-localization and MT-binding activity. CH: calponin homology domain, CC: coil-coiled domain, linker: the region between CH and CC. The numbers indicate the position of amino acids.

b IFA of GFP-tagged EB1 proteins (green) and MTs (red) in the human cell line MRC5. The fragment of PyEB1-N+M (1-307 aa) was fused with GFP at the C-terminal and transiently expressed in the human MRC5 cell line. Representative for two independent experiments. Scale bar = 5 μm.

c *in vitro* MT binding analysis of the PyEB1-N+M fragment by total internal reflection fluorescence (TIRF) microscopy. The GMPCPP-stabilized MT was used as nucleation seed for MT growth in the presence of GFP-tagged PyEB1-N+M (60 nM used). Representative images and kymographs showed the full-length MT binding of PyEB1-N+M. Note that MT growth *in vitro* occurs in both MT-plus and -minus ends. Representative for two independent experiments. Horizontal scale bars: 2 μm; vertical scale bars: 1 min.

a

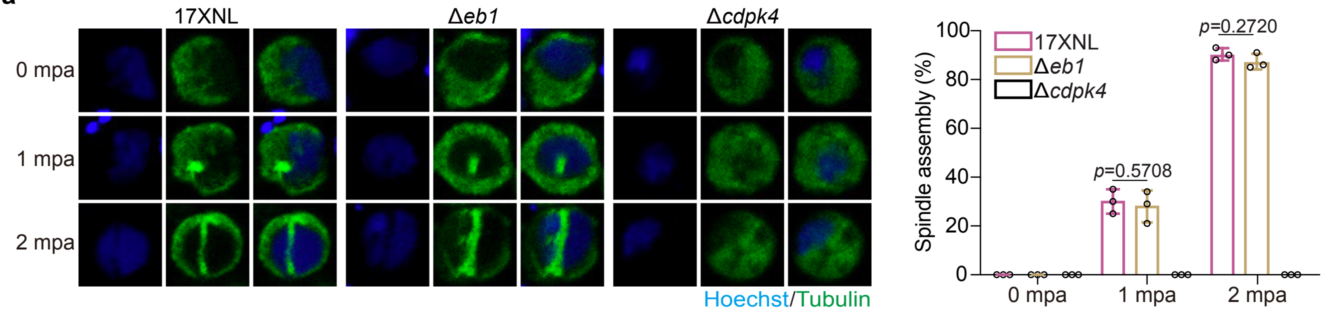
17XNL
Δeb1
Δcdpk4
0 mpa
1 mpa
2 mpa
Hoechst/Tubulin
Spindle assembly (%)
100
80
60
40
20
0
17XNL
Δeb1
Δcdpk4
p=0.5708
p=0.2720
0 mpa
1 mpa
2 mpa

Supplementary Figure 7. EB1 disruption does not affect spindle formation in early male gametogenesis

a IFA detection of intranuclear spindle in early male gametogenesis (0, 1, and 2 mpa) of 17XNL and Δ*eb1* parasites. Parasites were co-stained with antibody against both α-Tubulin I and α-Tubulin II (male gametocyte marker) and Hoechst 33342. The parasite mutant with genetic deletion of *cdpk4*, an essential gene for endomitosis in male gametogenesis, was used as a negative control. Scale bar = 5 µm. Right panel indicates the percentage of male gametocytes showing early nuclear spindle. Mean ± SD from three independent experiments, two-tailed t-test.

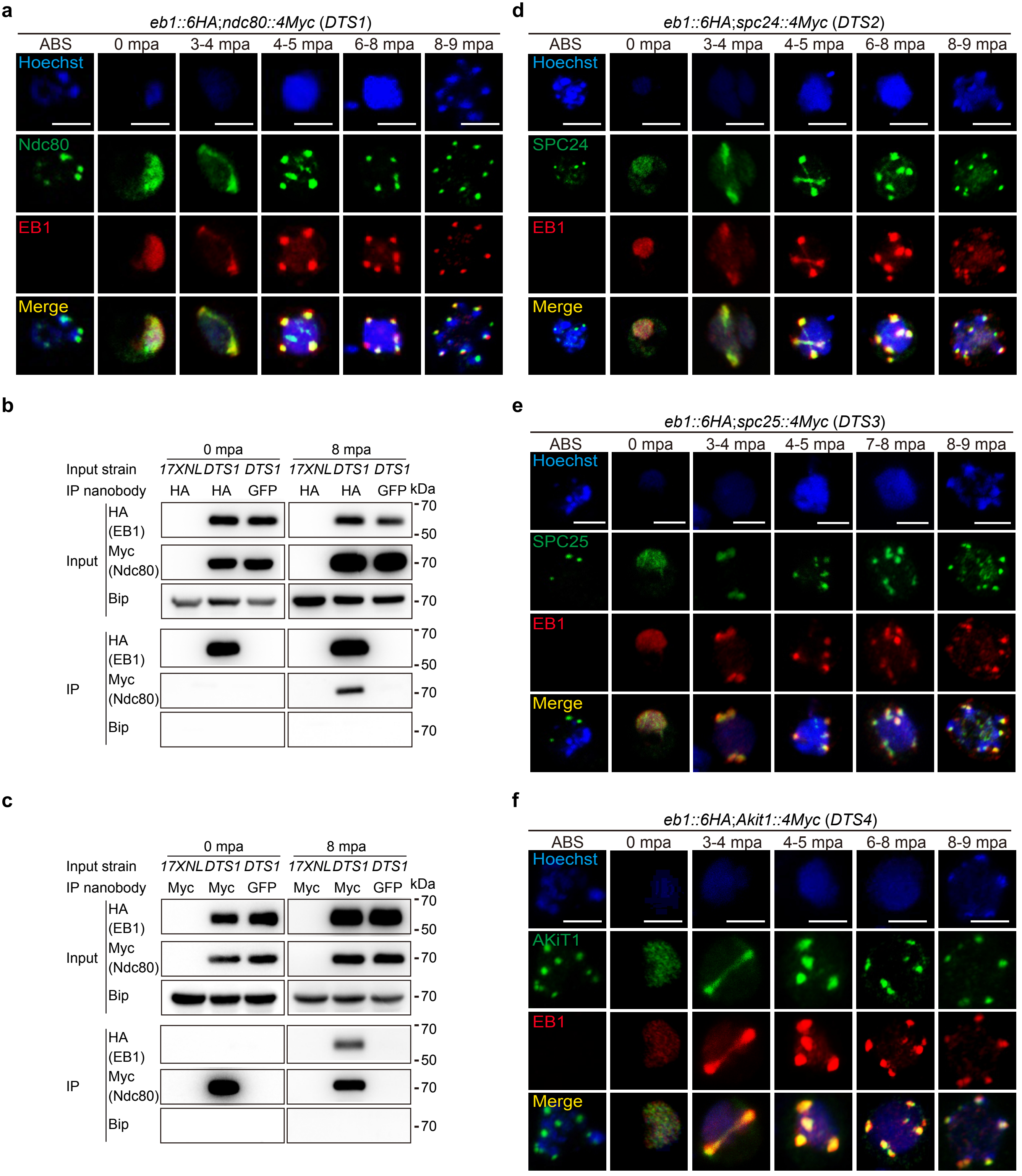
a
eb1::6HA;ndc80::4Myc (DTS1)
ABS
0 mpa
3-4 mpa
4-5 mpa
6-8 mpa
8-9 mpa
Hoechst
Ndc80
EB1
Merge
b
0 mpa
8 mpa
Input strain
17XNL DTS1 DTS1
17XNL DTS1 DTS1
IP nanobody
HA HA GFP
HA HA GFP
kDa
Input
HA (EB1)
Myc (Ndc80)
Bip
IP
HA (EB1)
Myc (Ndc80)
Bip
70
50
70
70
70
50
70
70
c
0 mpa
8 mpa
Input strain
17XNL DTS1 DTS1
17XNL DTS1 DTS1
IP nanobody
Myc Myc GFP
Myc Myc GFP
kDa
Input
HA (EB1)
Myc (Ndc80)
Bip
IP
HA (EB1)
Myc (Ndc80)
Bip
70
50
70
70
70
50
70
70
d
eb1::6HA;spc24::4Myc (DTS2)
ABS
0 mpa
3-4 mpa
4-5 mpa
6-8 mpa
8-9 mpa
Hoechst
SPC24
EB1
Merge
e
eb1::6HA;spc25::4Myc (DTS3)
ABS
0 mpa
3-4 mpa
4-5 mpa
7-8 mpa
8-9 mpa
Hoechst
SPC25
EB1
Merge
f
eb1::6HA;Akit1::4Myc (DTS4)
ABS
0 mpa
3-4 mpa
4-5 mpa
6-8 mpa
8-9 mpa
Hoechst
AKiT1
EB1
Merge

Supplementary Figure 8. EB1-decorated spindles co-localize and interact with kinetochores throughout male gametogenesis

a IFA of EB1 and Ndc80 (kinetochore protein) in asexual blood stages and during male gametogenesis of a double-tagged parasite strain *eb1::6HA*;*ndc80::4Myc* (*DTS1*). The parasites were co-stained with the antibodies against HA and Myc and Hoechst 33342. Representative for three independent experiments. Scale bar = 5 μm.

b Co-immunoprecipitation of EB1 and Ndc80 in the *DTS1* gametocytes at 0 and 8 mpa. The anti-HA nanobody was used for immunoprecipitation. BiP as loading control. Representative for two independent experiments.

c Co-immunoprecipitation of EB1 and Ndc80 in the *DTS1* gametocytes at 0 and 8 mpa. The anti-Myc nanobody was used for immunoprecipitation. BiP as loading control. Representative for two independent experiments.

d IFA of EB1 and SPC24 (kinetochore protein) in asexual blood stages and during male gametogenesis of a double-tagged parasite strain *eb1::6HA*;*spc24::4Myc* (*DTS2*). The parasites were co-stained with the antibodies against HA and Myc and Hoechst 33342. Representative for three independent experiments. Scale bar = 5 μm.

e IFA of EB1 and SPC25 (kinetochore protein) in asexual blood stages and during male gametogenesis of a double-tagged parasite strain *eb1::6HA*;*spc25::4Myc* (*DTS3*). The parasites were co-stained with the antibodies against HA and Myc and Hoechst 33342. Representative for three independent experiments. Scale bar = 5 μm..

f IFA of EB1 and AKiT1 (kinetochore protein) in asexual blood stages and during male gametogenesis of a double-tagged parasite strain *eb1::6HA*;*akit1::4Myc* (*DTS4*). The parasites were co-stained with the antibodies against HA and Myc and Hoechst 33342. Representative for three independent experiments. Scale bar = 5 μm.

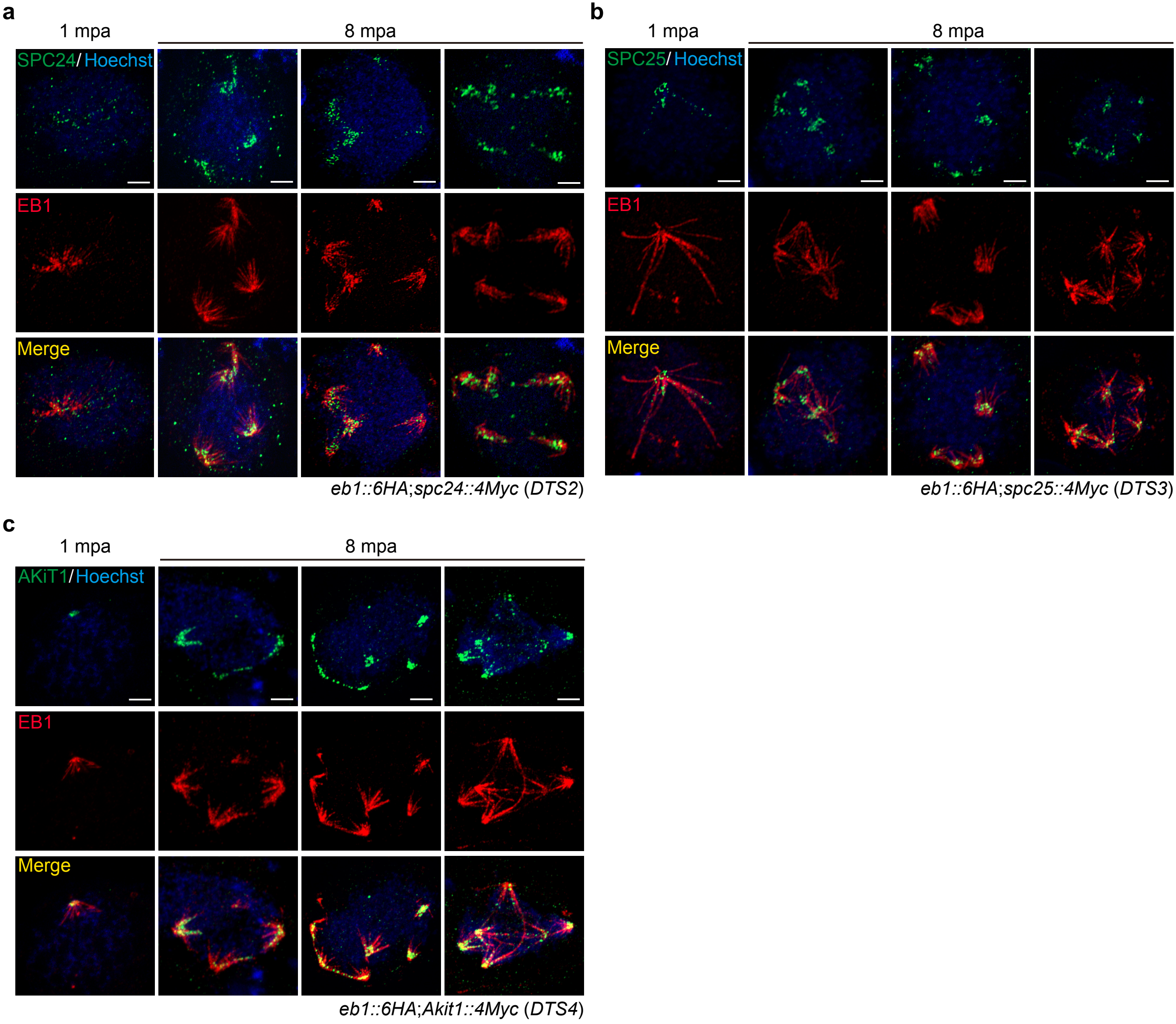
a
1 mpa
8 mpa
SPC24/ Hoechst
EB1
Merge
eb1::6HA;spc24::4Myc (DTS2)
b
1 mpa
8 mpa
SPC25/ Hoechst
EB1
Merge
eb1::6HA;spc25::4Myc (DTS3)
c
1 mpa
8 mpa
AKiT1/ Hoechst
EB1
Merge
eb1::6HA;Akit1::4Myc (DTS4)

Supplementary Figure 9. Kinetochores connect spindle laterally during male gametogenesis

a U-ExM of EB1 and SPC24 during male gametogenesis of the double-tagged strain *eb1::6HA*;*spc24::4Myc* (*DTS2*). Parasites were co-stained with the antibodies against HA and Myc epitopes. Representative for three independent experiments. Scale bar = 5 μm.

b U-ExM of EB1 and SPC25 during male gametogenesis of the double-tagged strain *eb1::6HA*;*spc25::4Myc* (*DTS3*). Parasites were co-stained with the antibodies against HA and Myc epitopes. Representative for three independent experiments. Scale bar = 5 μm.

c U-ExM of EB1 and AKiT1 during male gametogenesis of the double-tagged strain *eb1::6HA*;*Akit1::4Myc* (*DTS4*). Parasites were co-stained with the antibodies against HA and Myc epitopes. Representative for three independent experiments. Scale bar = 5 μm

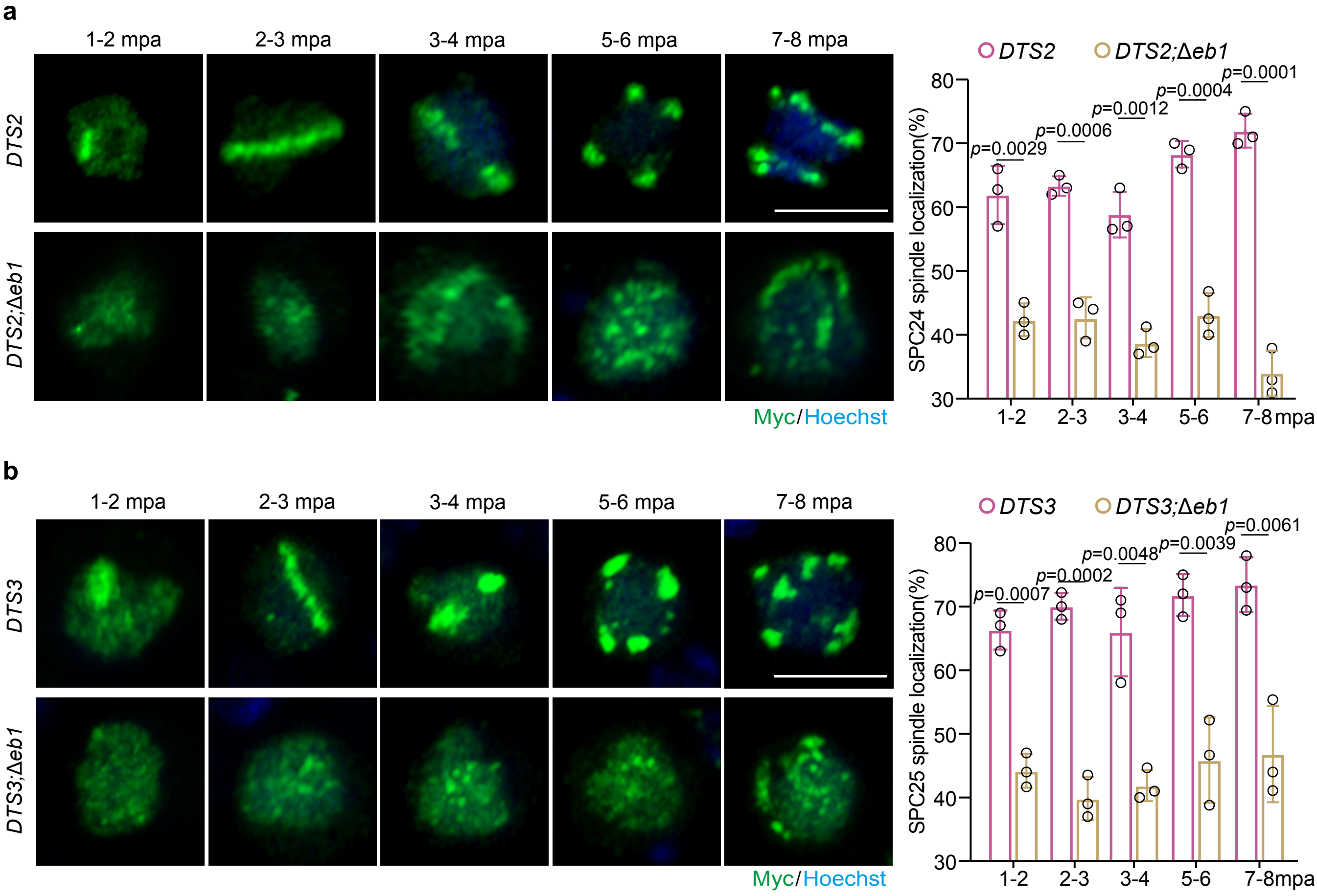
a
1-2 mpa
2-3 mpa
3-4 mpa
5-6 mpa
7-8 mpa
DTS2
DTS2;Δeb1
Myc/Hoechst
DTS2
DTS2;Δeb1
SPC24 spindle localization(%)
p=0.0029
p=0.0006
p=0.0012
p=0.0004
p=0.0001
80
70
60
50
40
30
1-2
2-3
3-4
5-6
7-8 mpa
b
1-2 mpa
2-3 mpa
3-4 mpa
5-6 mpa
7-8 mpa
DTS3
DTS3;Δeb1
Myc/Hoechst
DTS3
DTS3;Δeb1
SPC25 spindle localization(%)
p=0.0007
p=0.0002
p=0.0048
p=0.0039
p=0.0061
80
70
60
50
40
30
1-2
2-3
3-4
5-6
7-8mpa

Supplementary Figure 10. The spindle-kinetochore attachment is dependent on EB1

a IFA of SPC24 during male gametogenesis of the *eb1::6HA*;*spc24::4Myc* (*DTS2*) and *DTS2*;Δ*eb1* parasites. *DTS2*;Δ*eb1* is a *DTS2*-derived EB1-null strain. The parasites were co-stained with Hoechst 33342 and anti-Myc antibody. Scale bar = 5 μm. Right panel indicates the percentage of male gametocytes showing spindle localization of SPC24. Mean ± SD from three independent experiments, two-tailed t-test.

b IFA of SPC25 during male gametogenesis of the *eb1::6HA*;*spc25::4Myc* (*DTS3*) and *DTS3*;Δ*eb1* parasites. *DTS3*;Δ*eb1* is a *DTS3*-derived EB1-null strain. The parasites were co-stained with Hoechst 33342 and anti-Myc antibody. Scale bar = 5 μm. Right panel indicates the percentage of male gametocytes showing spindle localization of SPC25. Mean ± SD from three independent experiments, two-tailed t-test.

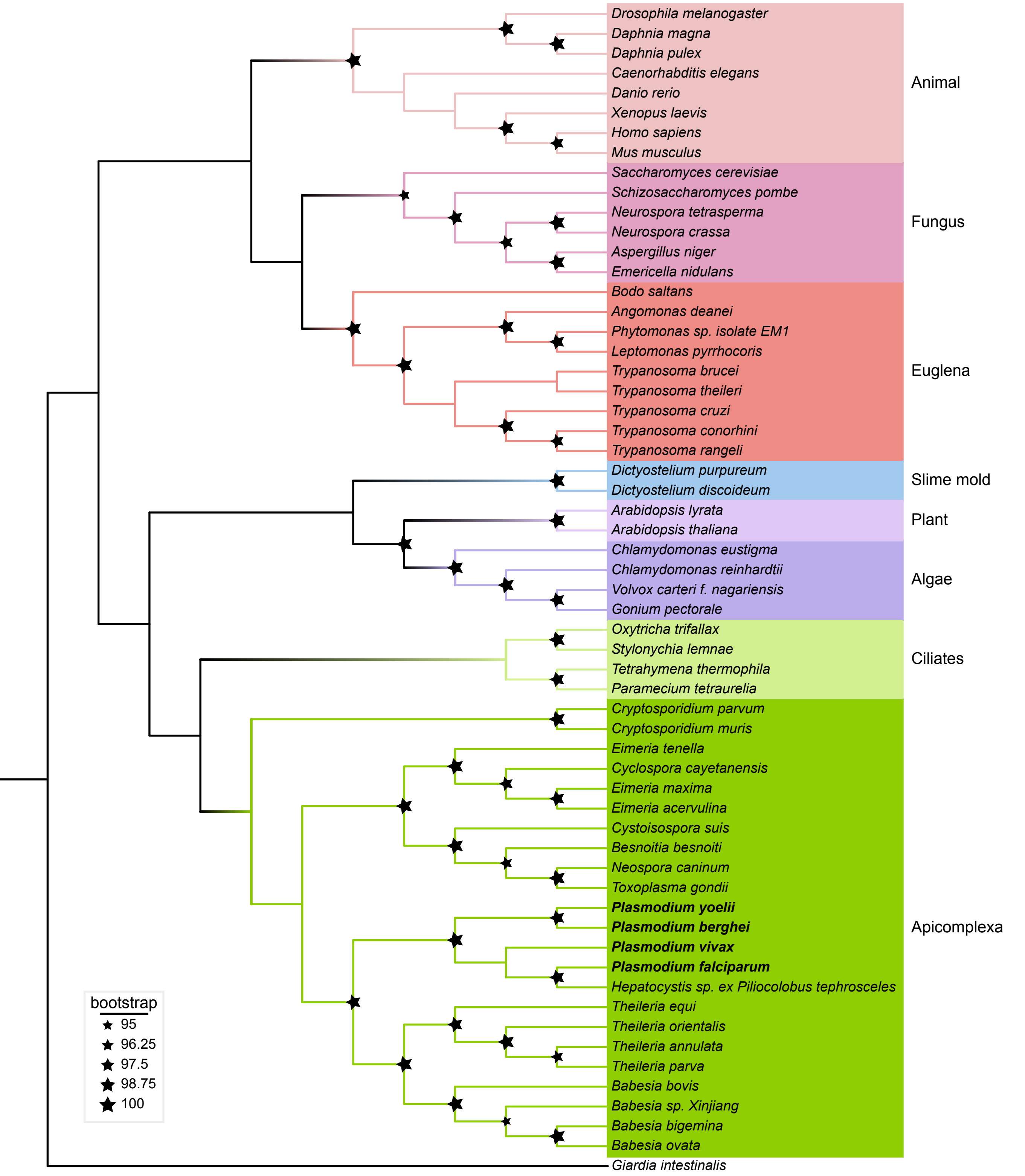
Drosophila melanogaster
Daphnia magna
Daphnia pulex
Caenorhabditis elegans
Danio rerio
Xenopus laevis
Homo sapiens
Mus musculus
Animal
Saccharomyces cerevisiae
Schizosaccharomyces pombe
Neurospora tetrasperma
Neurospora crassa
Aspergillus niger
Emericella nidulans
Fungus
Bodo saltans
Angomonas deanei
Phytomonas sp. isolate EM1
Leptomonas pyrrhocoris
Trypanosoma brucei
Trypanosoma theileri
Trypanosoma cruzi
Trypanosoma conorhini
Trypanosoma rangeli
Euglena
Dictyostelium purpureum
Dictyostelium discoideum
Slime mold
Arabidopsis lyrata
Arabidopsis thaliana
Plant
Chlamydomonas eustigma
Chlamydomonas reinhardtii
Volvox carteri f. nagariensis
Gonium pectorale
Algae
Oxytricha trifallax
Stylonychia lemnae
Tetrahymena thermophila
Paramecium tetraurelia
Ciliates
Cryptosporidium parvum
Cryptosporidium muris
Eimeria tenella
Cyclospora cayetanensis
Eimeria maxima
Eimeria acervulina
Cystoisospora suis
Besnoitia besnoiti
Neospora caninum
Toxoplasma gondii
Plasmodium yoelii
Plasmodium berghei
Plasmodium vivax
Plasmodium falciparum
Hepatocystis sp. ex Piliocolobus tephrosceles
Theileria equi
Theileria orientalis
Theileria annulata
Theileria parva
Babesia bovis
Babesia sp. Xinjiang
Babesia bigemina
Babesia ovata
Apicomplexa
Giardia intestinalis
bootstrap
95
96.25
97.5
98.75
100

Supplementary Figure 11. Phylogenetic analysis of EB1 protein from the eukaryotic species

Phylogenetic tree of EB1 homologs from different eukaryotic species. Multiple sequence alignment of EB1 homologs from species in different taxonomies was used to generate the phylogenetic tree. The size of star indicates ultrafast bootstrap value. Taxonomies were marked with different background colors.

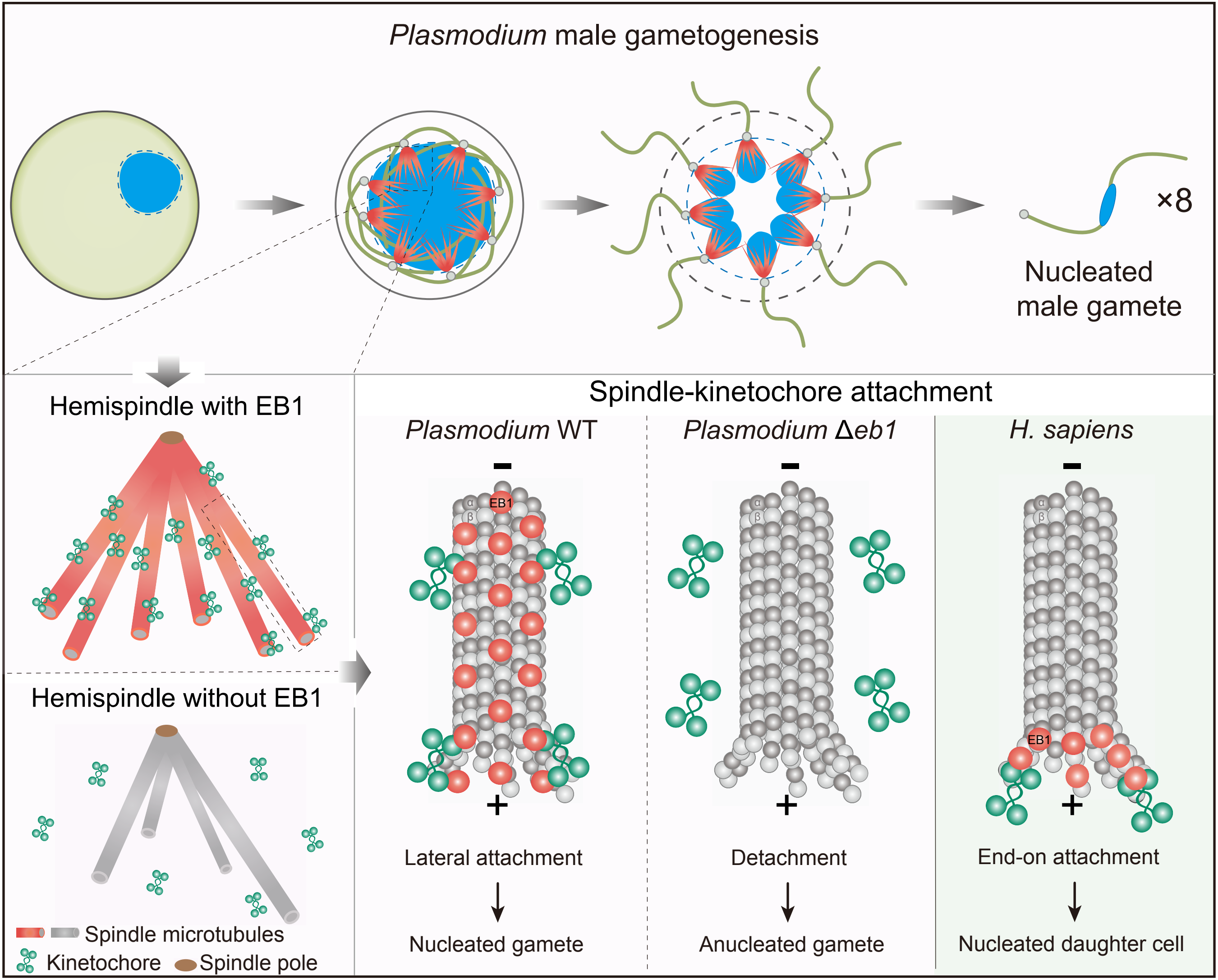

Plasmodium male gametogenesis
×8
Nucleated male gamete
Hemispindle with EB1
Hemispindle without EB1
Spindle microtubules
Kinetochore
Spindle pole
Spindle-kinetochore attachment
Plasmodium WT
Plasmodium Δ*eb1*
H. sapiens
EB1
α
β
Lateral attachment
Nucleated gamete
Detachment
Anucleated gamete
End-on attachment
Nucleated daughter cell

Supplementary Figure 12. A proposed model for the unique MT-lattice binding EB1 in spindle-kinetochore lateral attachment during *Plasmodium* male gametogenesis

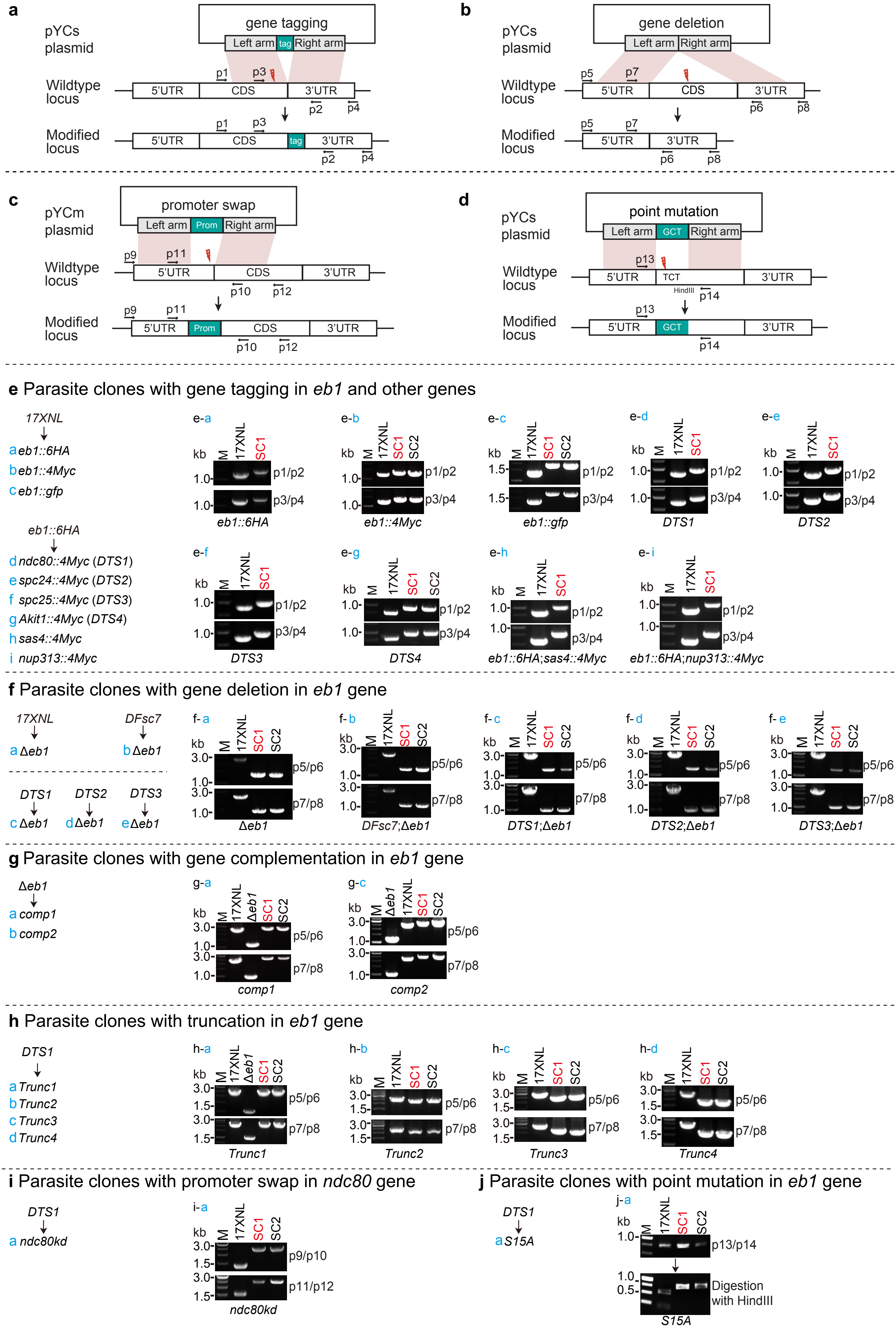
a
pYCs plasmid
gene tagging
Left arm tag Right arm
Wildtype locus
5'UTR CDS 3'UTR
p1 p3 p2 p4
Modified locus
5'UTR CDS tag 3'UTR
b
pYCs plasmid
gene deletion
Left arm Right arm
Wildtype locus
5'UTR CDS 3'UTR
p5 p7 p6 p8
Modified locus
5'UTR 3'UTR
c
pYCm plasmid
promoter swap
Left arm Prom Right arm
Wildtype locus
5'UTR CDS 3'UTR
p9 p11 p10 p12
Modified locus
5'UTR Prom CDS 3'UTR
d
pYCs plasmid
point mutation
Left arm GCT Right arm
Wildtype locus
5'UTR TCT 3'UTR
HindIII
p13 p14
Modified locus
5'UTR GCT 3'UTR
e Parasite clones with gene tagging in eb1 and other genes
17XNL
a eb1::6HA
b eb1::4Myc
c eb1::gfp
eb1::6HA
d ndc80::4Myc (DTS1)
e spc24::4Myc (DTS2)
f spc25::4Myc (DTS3)
g Akit1::4Myc (DTS4)
h sas4::4Myc
i nup313::4Myc
e-a e-b e-c e-d e-e e-f e-g e-h e-i
kb M 17XNL SC1 SC2
1.0 1.5
p1/p2 p3/p4
eb1::6HA
eb1::4Myc
eb1::gfp
DTS1
DTS2
DTS3
DTS4
eb1::6HA;sas4::4Myc
eb1::6HA;nup313::4Myc
f Parasite clones with gene deletion in eb1 gene
17XNL
a Δeb1
DFsc7
b Δeb1
DTS1
c Δeb1
DTS2
d Δeb1
DTS3
e Δeb1
f-a f-b f-c f-d f-e
kb M 17XNL SC1 SC2
3.0 1.0
p5/p6 p7/p8
Δeb1
DFsc7;Δeb1
DTS1;Δeb1
DTS2;Δeb1
DTS3;Δeb1
g Parasite clones with gene complementation in eb1 gene
Δeb1
a comp1
b comp2
g-a g-c
kb M 17XNL Δeb1 SC1 SC2
3.0 1.0
p5/p6 p7/p8
comp1
comp2
h Parasite clones with truncation in eb1 gene
DTS1
a Trunc1
b Trunc2
c Trunc3
d Trunc4
h-a h-b h-c h-d
kb M 17XNL Δeb1 SC1 SC2
3.0 1.5
p5/p6 p7/p8
Trunc1
Trunc2
Trunc3
Trunc4
i Parasite clones with promoter swap in ndc80 gene
DTS1
a ndc80kd
i-a
kb M 17XNL SC1 SC2
3.0 1.5
p9/p10 p11/p12
ndc80kd
j Parasite clones with point mutation in eb1 gene
DTS1
a S15A
j-a
kb M 17XNL SC1 SC2
1.0 0.5
p13/p14
Digestion with HindIII
S15A

Supplementary Figure 13. Genotyping of genetically modified parasites in this study
a-d Schematic representation for CRISPR-Cas9 mediated gene modification, including gene tagging (A), gene deletion (B), promoter swap (C), and point mutation (D) via double-cross homologous recombination.
e-j For each modification, both 5' and 3' homologous recombination were detected by the genotyping PCR (see the primers in the Supplementary Table 1) to confirm successful integration of the homologous templates. Two independent experiments with similar results. Usually, 1-2 parasite single clones (sc) were obtained after limiting dilution, and one clone (indicated with red letter) was used for further phenotype and gene functional analysis.

Table S1. Primers and oligonucleotides used in this study

**Primers for gene knockout**									
**Gene name**	**Gene ID**	**Modified strain**	**Gene size /deleted gene size (bp)**	**Left homologous template**		**Right homologous template**		**Target site of sgRNA**	
				**Forward primer**	**Reverse primer**	**Forward primer**	**Reverse primer**	**Oligo (Forward)**	**Oligo (Reverse)**
eb1	PY17X_0407900	*Δeb1*	1293/1293	CGGGGTACCCATTAATTTCGACAATTTTAAGAT	CATGCCATGGAATTTATTTTTTTCTTCTCAGCTTTT	CCGCTCGAGCTACACTTATCTTGATTAACCCT	CCCCTTAAGTGGGGGTTTATGTTTTAACT	TATTAATTGGGGATATTATTCTGT	AAACACAGAATAATATCCCCAATT
eb1	PY17X_0407900	*Trunc1*	1293/54	CGGGGTACCCATTAATTTCGACAATTTTAAGAT	CATGCCATGGAATTTATTTTTTTCTTCTCAGCTTTT	CCGCTCGAGCTACACTTATCTTGATTAACCCT	CCCCTTAAGTGGGGGTTTATGTTTTAACT	TATTGCGGACGCTAATCGTAGCTA	AAACTAGCTACGATTAGCGTCCGC
eb1	PY17X_0407900	*Trunc2*	1293/459	CGGGGTACCCATTAATTTCGACAATTTTAAGAT	CATGCCATGGAAATCCAGAATCCATGTTTC	CATGCCATGGAAAATAATTTCAAATAGCTC	CCGCTCGAGATCTTTTACATTTTCTGAAATGC	TATTATAACTTAGAATTCCTACAA	AAACTTGTAGGAATTCTAAGTTAT
eb1	PY17X_0407900	*Trunc3*	1293/414	CGGGGTACCGTGCTAAATGAGCTTGTGAA	CCGCTCGAGTTTTATAATATTTAAATCAACTTTTGCC	CCGCTCGAGAATATATCTTTAATTGAACAAAATAAAAAATT	CCCCTTAAGCACACACACACACAATATAT	TATTAATTGGGGATATTATTCTGT	AAACACAGAATAATATCCCCAATT
eb1	PY17X_0407900	*Trunc4*	1293/870	CGGGGTACCCATTAATTTCGACAATTTTAAGAT	CCGCTCGAGAAATCCAGAATCCATGTTTC	CCGCTCGAGAATATATCTTTAATTGAACAAAATAAAAAATT	CCCCTTAAGCACACACACACACAATATAT	TATTAATTGGGGATATTATTCTGT	AAACACAGAATAATATCCCCAATT
**Primers for PCR-genotyping parasite with gene knockout**									
**Gene name**	**Gene ID**	**Modified strain**	**P1**	**P2**	**P3**	**P4**	**CDS(19-431)-6HA- Forward**	**CDS(19-431)-6HA- Reverse**	
eb1	PY17X_0407900	*Δeb1*	GGATACATAAGTATGTCCTCGT	TGGGGGTTTATGTTTTAACT	CATTAATTTCGACAATTTTAAGAT	ACCTAACCGAGAATTGATAT			
eb1	PY17X_0407900	*Trunc1*	GGATACATAAGTATGTCCTCGT	TGGGGGTTTATGTTTTAACT	CATTAATTTCGACAATTTTAAGAT	ACCTAACCGAGAATTGATAT	CATGCCATGGGGGTAAGTCGAAAAGAACTA	CCGCTCGAGTGATCGTTCCACTTTTTA	
eb1	PY17X_0407900	*Trunc2*	GGATACATAAGTATGTCCTCGT	TGGGGGTTTATGTTTTAACT	CATTAATTTCGACAATTTTAAGAT	ACCTAACCGAGAATTGATAT			
eb1	PY17X_0407900	*Trunc3*	GGATACATAAGTATGTCCTCGT	TGGGGGTTTATGTTTTAACT	CATTAATTTCGACAATTTTAAGAT	ACCTAACCGAGAATTGATAT			
eb1	PY17X_0407900	*Trunc4*	GGATACATAAGTATGTCCTCGT	TGGGGGTTTATGTTTTAACT	CATTAATTTCGACAATTTTAAGAT	ACCTAACCGAGAATTGATAT			
**Primers for gene complementation**									
**Gene name**	**Gene ID**	**Modified strain**	**Gene size /re-introduced gene size (bp)**	**Left homologous template**		**Right homologous template**		**Target site of sgRNA**	
				**Forward primer**	**Reverse primer**	**Forward primer**	**Reverse primer**	**Oligo (Forward)**	**Oligo (Reverse)**
Pyeb1	PY17X_0407900	*comp1*	1293/1290	CGGGGTACCCATTAATTTCGACAATTTTAAGAT	CATGCCATGGAATTTATTTTTTTCTTCTCAGCTTTT	CCGCTCGAGCTACACTTATCTTGATTAACCCT	CCCCTTAAGTGGGGGTTTATGTTTTAACT	TATTGCGGACGCTAATCGTAGCTA	AAACTAGCTACGATTAGCGTCCGC
**Gene name**	**Gene ID**	**Modified strain**	**Gene size /re-introduced gene size (bp)**	**CDS**		**C6HA**		**CDS-C6HA**	
				**Forward primer**	**Reverse primer**	**Forward primer**	**Reverse primer**	**Forward primer**	**Reverse primer**
Pyeb1	PY17X_0407900	*comp1*	1293/1290	CATGCCATGGATGCATGAAGAGAAAGAAACATTA	CAGAACCGGATTCTGTAGGCAAATTGATGG	GCCTACAGAAtCCGGTTCTGCTGCTAGATA	CCGCTCGAGTGATCGTTCCACTTTTTAGCTAGA	CATGCCATGGATGCATGAAGAGAAAGAAACATTA	CCGCTCGAGTGATCGTTCCACTTTTTAGCTAGA
**Gene name**	**Gene ID**	**Modified strain**	**Gene size /re-introduced gene size (bp)**	**Left homologous arm**		**Right homologous arm**		**Target site of sgRNA**	
				**Forward primer**	**Reverse primer**	**Forward primer**	**Reverse primer**	**Oligo (Forward)**	**Oligo (Reverse)**
Pfeb1	PF3D7_0307300	*comp2*	1533/1530	CGGGGTACCCATTAATTTCGACAATTTTAAGAT	CATGCCATGGAATTTATTTTTTTCTTCTCAGCTTTT	CCGCTCGAGCTACACTTATCTTGATTAACCCT	CCCCTTAAGTGGGGGTTTATGTTTTAACT	TATTGCGGACGCTAATCGTAGCTA	AAACTAGCTACGATTAGCGTCCGC
**Gene name**	**Gene ID**	**Modified strain**	**Gene size /re-introduced gene size (bp)**	**CDS**		**C6HA**		**CDS-C6HA**	
				**Forward primer**	**Reverse primer**	**Forward primer**	**Reverse primer**	**Forward primer**	**Reverse primer**
Pfeb1	PF3D7_0307300	*comp2*	1533/1530	CATGCCATGGATGACTGAATATAAAGACTTAC	CAGAACCGGATGAACAGTAGGTGGCGTAGT	CTACTGTTCAtCCGGTTCTGCTGCTAGATA	CCGCTCGAGTGATCGTTCCACTTTTTAGCTAGA	CATGCCATGGATGACTGAATATAAAGACTTAC	CCGCTCGAGTGATCGTTCCACTTTTTAGCTAGA
**Primers for PCR-genotyping parasite with gene complementation**									
**Gene name**	**Gene ID**	**Modified strain**	**Gene size /re-introduced gene size (bp)**	**P1**	**P2**	**P3**	**P4**		
Pyeb1	PY17X_0407900	*comp1*	1293/1290	GGATACATAAGTATGTCCTCGT	TGGGGGTTTATGTTTTAACT	CATTAATTTCGACAATTTTAAGAT	ACCTAACCGAGAATTGATAT		
Pfeb1	PF3D7_0307300	*comp2*	1533/1530	GGATACATAAGTATGTCCTCGT	TGGGGGTTTATGTTTTAACT	CATTAATTTCGACAATTTTAAGAT	ACCTAACCGAGAATTGATAT		
**Primers for gene tagging**									
**Gene name**	**Tag (name and location)**	**Modified strain**	**Gene ID**	**Left homologous template**		**Right homologous template**		**Target site of sgRNA**	
				**Forward primer**	**Rerverse primer**	**Forward primer**	**Reverse primer**	**Oligo (Forward)**	**Oligo (Reverse)**
eb1	C-terminal 6HA	*eb1::6HA*	PY17X_0407900	CGGGGTACCCAACAATTGTGAATGATACAAC	CATGCCATGGTTCTGTAGGCAAATTGATGG	CCGCTCGAGCTACACTTATCTTGATTAACCCT	CCCCTTAAGTGGGGGTTTATGTTTTAACT	TATTAATTGGGGATATTATTCTGT	AAACACAGAATAATATCCCCAATT
eb1	C-terminal 4Myc	*eb1::4Myc*	PY17X_0407900	CGGGGTACCCAACAATTGTGAATGATACAAC	CATGCCATGGTTCTGTAGGCAAATTGATGG	CCGCTCGAGCTACACTTATCTTGATTAACCCT	CCCCTTAAGTGGGGGTTTATGTTTTAACT	TATTAATTGGGGATATTATTCTGT	AAACACAGAATAATATCCCCAATT
eb1	C-terminal GFP	*eb1::GFP*	PY17X_0407900	CGGGGTACCCAACAATTGTGAATGATACAAC	CATGCCATGGTTCTGTAGGCAAATTGATGG	CCGCTCGAGCTACACTTATCTTGATTAACCCT	CCCCTTAAGTGGGGGTTTATGTTTTAACT	TATTAATTGGGGATATTATTCTGT	AAACACAGAATAATATCCCCAATT
ndc80	C-terminal 4Myc	*ndc80::4Myc*	PY17X_1116900	CCCAAGCTTATTGGGACGCTAATTAGCGA	CATGCCATGGTTCAGTTACACTTTGGTGTA	CCGCTCGAGATATAAGCAAATAAAAAATGTGC	CCGGAATTCGAAGATCAAACAACAGATGC	TATTTTTATATTACATCAGGGTGA	AAACTCACCCTGATGTAATATAAA
spc24	C-terminal 4Myc	*spc24::4Myc*	PY17X_1444800	CGGGGTACCGTGGAGAAAGTGATATTGATGAT	CATGCCATGGAAAATAATTATCAAAAGTTTCTTTGT	CCGCTCGAGGCATGCCAACCATTCTAAAA	CCCCTTAAGGGCATATTCTTTTCTTTTGTATATCTTC	TATTCAGAAGCATGTGATTATTTA	AAACTAAATAATCACATGCTTCTG
spc25	C-terminal 4Myc	*spc25::4Myc*	PY17X_1364500	CGGGGTACCGAATGGACGAAGAAATTGGAAA	CATGCCATGGTTGTAACTCTTTAAATGATTTTCTGAGC	CCGCTCGAGATTCTAATAAAAATGTGTCCATCAG	CCCCTTAAGGAATCGGCATTTTCTAGGCA	TATTTATGTAAATGGGTTGAATAG	AAACCTATTCAACCCATTTACATA
Akit1	C-terminal 4Myc	*Akit1::4Myc*	PY17X_0624000	CGGGGTACCAAGGTAGAAAGCGTACATG	CATGCCATGGTTCAGAAATTTTTTCTTCGATTCTTT	CCGCTCGAGGTTGGCCTCGCACGTGTATC	CCCCTTAAGCGTGACCCTAAACAGTTTATGCAG	TATTTTGAGCGAACTCGCGATATT	AAACAATATCGCGAGTTCGCTCAA
sas4	C-terminal 4Myc	*sas4::4Myc*	PY17X_1326000	CGGGGTACCCATGCTTGTTCCTCAATTCA	CATGCCATGGATTTAACCTTTTTATGACACCT	CCGCTCGAGAACACTAAATATAATTAACAAATATTCTCG	CCCCTTAAGCCAAACAATGGCTAACAATT	TATTTATGGGAGCCAATAAAAAGG	AAACCCTTTTTATTGGCTCCCATA
nup313	C-terminal 4Myc	*nup313::4Myc*	PY17X_1314100	CGGGGTACCGGCGATCTATTATGGATGATGT	CATGCCATGGGTTTAAGACATTTTGGCTAGTAAA	CCGCTCGAGTTACATTCAACATGTCTTACCA	CCCCTTAAGCCCTTTCTTTGTTCCAAATTAATG	TATTTGTGTGTGTGTGCAAGTCAA	AAACTTGACTTGCACACACACACA
**Primer sequence for PCR-genotyping parasite with gene tagging**									
**Gene name**	**Tag (name and location)**	**Modified strain**	**Gene ID**	**P1**	**P2**	**P3**	**P4**		
eb1	C-terminal 6HA	*eb1::6HA*	PY17X_0407900	CAGCCAAATCAACAACAAT	TGGGGGTTTATGTTTTAACT	CAACAATTGTGAATGATACAAC	ACCTAACCGAGAATTGATAT		
eb1	C-terminal 4Myc	*eb1::4Myc*	PY17X_0407900	CAGCCAAATCAACAACAAT	TGGGGGTTTATGTTTTAACT	CAACAATTGTGAATGATACAAC	ACCTAACCGAGAATTGATAT		
eb1	C-terminal GFP	*eb1::GFP*	PY17X_0407900	CAGCCAAATCAACAACAAT	TGGGGGTTTATGTTTTAACT	CAACAATTGTGAATGATACAAC	ACCTAACCGAGAATTGATAT		
ndc80	C-terminal 4Myc	*ndc80::4Myc*	PY17X_1116900	AGAATGAGGCAAATGAAATG	GATATGCATACATATGTATAC	AGGGTGAAGGTATTACTATACA	AAAAATGAATTCTCCCTTTG		
spc24	C-terminal 4Myc	*spc24::4Myc*	PY17X_1444800	CCAGGACGATTCATCTTACTTTAA	TTTTTTAGAATGGTTGGCATGC	AAACGAACCTATCTACTCATTG	GCATAATTATTACATATGCACAAGTC		
spc25	C-terminal 4Myc	*spc25::4Myc*	PY17X_1364500	GCAGAATATAAAACCGTTAAACTGC	CAATTTCGTTAGTCACAAAC	CATACACACAAATATAAAGGAA	ATACACCCGACTAATCTTTG		
Akit1	C-terminal 4Myc	*Akit1::4Myc*	PY17X_0624000	AAATGTCGAGGAGTGCTTTT	ATGACCAAGCATAATAAACA	ACAACTTTCACACATATCAAA	AGCCTAGATGGTATGTTTGC		
sas4	C-terminal 4Myc	*sas4::4Myc*	PY17X_1326000	CTTCCCTAATTCGTATACGG	TAATTCTCTCCAACTTAA	GGTGTCATAAAAAGGTTAAAT	ATATTTACGGCCATTCTACG		
nup313	C-terminal 4Myc	*nup313::4Myc*	PY17X_1314100	GCATGTTATTTTATGTACATGGT	TGGGTCTAATTCGAAAGTAT	CGAGAATTTTCATTAAGTCTT	TACATTCGCTTTTCAAAAGAG		
**Oligo sequences for *ndc80* promoter swap plasmid construction**									
**Gene name**	**Modification**	**Modified strain**	**Gene ID**	**Left homologous arm**		**Right homologous arm**		**Target site of sgRNA**	
				**Forward primer**	**Rerverse primer**	**Forward primer**	**Reverse primer**	**Oligo (Forward)**	**Oligo (Reverse)**
ndc80	promoter swap	*ndc80kd*	PY17X_1116900	CGGGGTACCCCCTCCTTGTTTTTAAAACTTA	CATGCCATGGGTTTAATGATTACACTAATT	CCGCTCGAGATGAATAACCATTCGGTGTA	CCCCTTAAGTATTTCACAATCTTCGTT	TATTAAGGGGTATTCGCCAATATG	AAACCATATTGGCGAATACCCCTT
**Primers for PCR-genotyping with *ndc80* promoter swap**									
**Gene name**	**Modification**	**Modified strain**	**Gene ID**	**P1**	**P2**	**P3**	**P4**		
ndc80	promoter swap	*ndc80kd*	PY17X_1116900	ACATATTTACAAGCTTCTCG	TACACCGAATGGTTATTCAT	GTGTGTAAATTAGTGTAATC	TTCTAACTTAGCACTAAGCA		
**Oligo sequences and Primers for *eb1* nucleotide replacement**									
**Gene name**	**Modified strain**	**Homologous arm**		**Mutation primers1(S15A)**		**Mutation primers2(HindIII)**		**Genotypic primers**	
		**Forward Primer**	**Reverse Primer**	**Forward Primer**	**Reverse Primer**	**Forward Primer**	**Reverse Primer**	**P1**	**P2**
eb1	*S15A*	CGGGGTACCTCCCCACATACATCCTTTTC	CATGCCATGGCAACTTTTGCCCAATCTG	ATTATCATTTGGAAACATGGATGCTGGATTTGTTGTAAGTCG	CTTTTCGACTTACAACAAATCCAGCATCCATGTTTCCAAATGA	TAGGAGAAAGAGGTGATTATAAACTTTTAAATAATTATATGCC	TCTGGCATATAATTATTTAAAAGTTTATAATCACCTCTTTCTC	GGATACATAAGTATGTCCTCGT	GTTCTTCATGTTAGTATTAG
**Primers for transient expression in HEK293T and MRC5**									
**Gene name**	**Gene ID**	**Description**	**CDS**				**Product length (bp)**		
			**Forward Primer**		**Reverse Primer**				
pyeb1	PY17X_0407900	PyEB1 FL-GFP-SII	ATTGCTAGCGCCACCATGCACGAGGAGAAGGAGAC		ATTAAGCTTCTCGGTGGGCAGGTTGAT		1290		
pyeb1	PY17X_0407900	SII-PyEB1 FL-GFP	GTTCGAAAAAGGCAGTCTAGCCACCATGCACGAGGAG		ATTAAGCTTCTCGGTGGGCAGGTTGAT		1290		
pyeb1	PY17X_0407900	PyEB1 1-177-LZ-GFP-SII N	ATTGCTAGCGCCACCATGCACGAGGAGAAGGAGAC		ATTAAGCTTGCTGCTGTTGCTGATGATCTT		532		
pyeb1	PY17X_0407900	SII-GFP-PyEB1 140-307 M	ATCAAGCTTGGAAGATCTGCATCCTGGGCG		ATCGGATCCTCAGTCCTTCACGTTCTCGCTGA		504		
pyeb1	PY17X_0407900	SII-GFP-PyEB1 307-430 C	ATCAAGCTTGGGACAACATCAGCCTGATCGAG		ATCGGATCCTCACTCGGTGGGCAGGTTGAT		373		
pyeb1	PY17X_0407900	PyEB1 1-307-LZ-GFP-SII N+M	ATTGCTAGCGCCACCATGCACGAGGAGAAGGAGAC		ATGGGATCCCCGTCCTTCACGTTCTCGCTGA		921		
pyeb1	PY17X_0407900	PyEB1 1-307-GFP-SII N+M	ATTGCTAGCGCCACCATGCACGAGGAGAAGGAGAC		ATGGGATCCCCGTCCTTCACGTTCTCGCTGA		921		
pyeb1	PY17X_0407900	PyEB1 FL-GFP-SII S15A	ATTGCTAGCGCCACCATGCACGAGGAGAAGGAGAC		ATTAAGCTTCTCGGTGGGCAGGTTGAT		1290		
hseb1	22919	HsEB1 FL-GFP-SII	TCCGCTAGCGCCACCATGGCAGTGAACGTATACTCAACG		CGGTGGATCCCCATACTCTTCTTGCTCCTCCTGTG		804		
hseb1	22919	HsEB1 FL-MCH-SII	TCCGCTAGCGCCACCATGGCAGTGAACGTATACTCAACG		CGGTGGATCCCCATACTCTTCTTGCTCCTCCTGTG		804		
hseb1	22919	HsEB1 1-137-LZ-GFP-SII N	TCCGCTAGCGCCACCATGGCAGTGAACGTATACTCAACG		ATCAAGCTTCACTGCAGTTTCTTGACCTTGT		411		
hseb1	22919	SII-GFP-HsEB1 131-268 C	ATCAAGCTTGGCAAGGTCAAGAAACTGCAGTGG		CGGTGGATCCTTAATACTCTTCTTGCTCCTCCTGTG		414		

Table S2. List of genetically modified parasite strains used in this study

**Strain**	**Description**			**Resource**
17XNL	*Plasmodium yoelii* 17XNL strain			NIH
**Parasite with gene deletion**	**Gene ID**	**Parental strain**	**Description**	**Resource**
Δ*eb1*	PY17X_0407900	17XNL	Deleted the whole coding sequence of *eb1* in the 17XNL parasite	This study
DFsc7; Δ*eb1*	PY17X_0407900	*DFsc7*	Deleted the whole coding sequence of *eb1* in the *DFsc7* parasite	This study
DTS1; Δ*eb1*	PY17X_0407900	*DTS1*	Deleted the whole coding sequence of *eb1* in the *DTS1* parasite	This study
DTS2; Δ*eb1*	PY17X_0407900	*DTS2*	Deleted the whole coding sequence of *eb1* in the *DTS2* parasite	This study
DTS3; Δ*eb1*	PY17X_0407900	*DTS3*	Deleted the whole coding sequence of *eb1* in the *DTS3* parasite	This study
Trunc1	PY17X_0407900	*DTS1*	Deleted the N-terminal part (1-18,18aa) of endogenous EB1	This study
Trunc2	PY17X_0407900	*DTS1*	Deleted the CH domain (18-170,153aa) of endogenous EB1	This study
Trunc3	PY17X_0407900	*DTS1*	Deleted the linker (170-307,138aa) of endogenous EB1	This study
Trunc4	PY17X_0407900	*DTS1*	Deleted the part including both CH domain and linker (18-307,290aa) of endogenous EB1	This study
**Gene compelementation strains**	**Gene ID**	**Parental strain**	**Description**	**Resource**
comp1	PY17X_0407900	Δ*eb1*	Complementation of HA tagged *pyeb1* sequence (1-1290 bp) in the Δ*eb1* parasite	This study
comp2	PF3D7_0307300	Δ*eb1*	Complementation of HA tagged *pfeb1* sequence (1-1530 bp) in the Δ*eb1* parasite	This study
**Parasites with gene tagging**	**Gene ID**	**Parental strain**	**Description**	**Resource**
eb1::6HA	PY17X_0407900	17XNL	C-terminally tagged with 6HA in the 17XNL parasite	This study
eb1::4Myc	PY17X_0407900	17XNL	C-terminally tagged with 4Myc in the 17XNL parasite	This study
eb1::GFP	PY17X_0407900	17XNL	C-terminally tagged with GFP in the 17XNL parasite	This study
eb1::6HA ; *ndc80::4Myc* (*DTS1*)	PY17X_1116900	*eb1::6HA*	C-terminally tagged with 4Myc in the *eb1::6HA* parasite	This study
eb1::6HA ; *spc24::4Myc* (*DTS2*)	PY17X_1444800	*eb1::6HA*	C-terminally tagged with 4Myc in the *eb1::6HA* parasite	This study
eb1::6HA ; *spc25::4Myc* (*DTS3*)	PY17X_1364500	*eb1::6HA*	C-terminally tagged with 4Myc in the *eb1::6HA* parasite	This study
eb1::6HA ; *Akit1::4Myc* (*DTS4*)	PY17X_0624000	*eb1::6HA*	C-terminally tagged with 4Myc in the *eb1::6HA* parasite	This study
eb1::6HA ; *sas4::4Myc*	PY17X_1326000	*eb1::6HA*	C-terminally tagged with 4Myc in the *eb1::6HA* parasite	This study
eb1::6HA ; *nup313::4Myc*	PY17X_1314100	*eb1::6HA*	C-terminally tagged with 4Myc in the *eb1::6HA* parasite	This study
**Parasites with promoter swap**	**Gene ID**	**Parental strain**	**Description**	**Resource**
ndc80kd	PY17X_1116900	*DTS1*	Replace 801 bp of endogenous *ndc80* promoter in the *DTS1* parasite with that (1826 bp) of *clag1* gene (PY17X_1402200)	This study
**Parasites with gene mutation**	**Gene ID**	**Parental strain**	**Description**	**Resource**
S15A	PY17X_0407900	*DTS1*	Replace the Serine 15 (S15) of the endogenous EB1 protein with Alanine (A) in the *DTS1* parasite	This study
