# Supplementary figures and images for: EB1 decoration of microtubule lattice facilitates spindle-kinetochore lateral attachment in Plasmodium male gametogenesis
Source: Nat Commun. 2023 May 19;14:2864. doi: 10.1038/s41467-023-38516-3 (PMC10199041; doi:10.1038/s41467-023-38516-3)

Fig. 1B

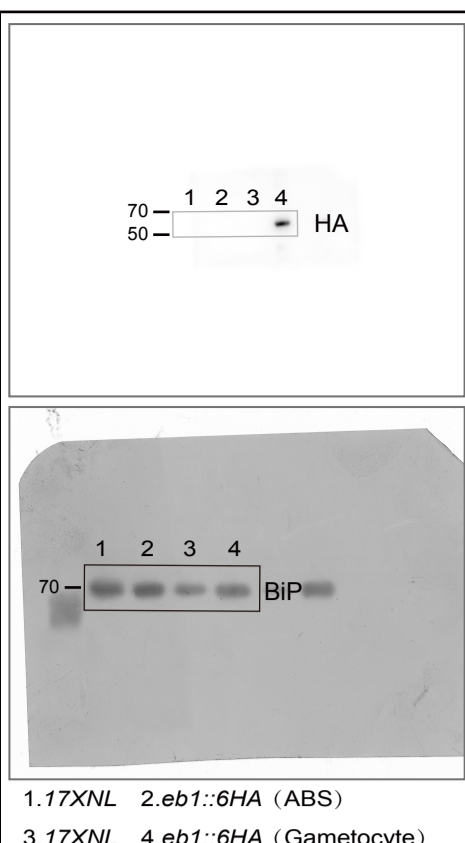

Fig. 1D

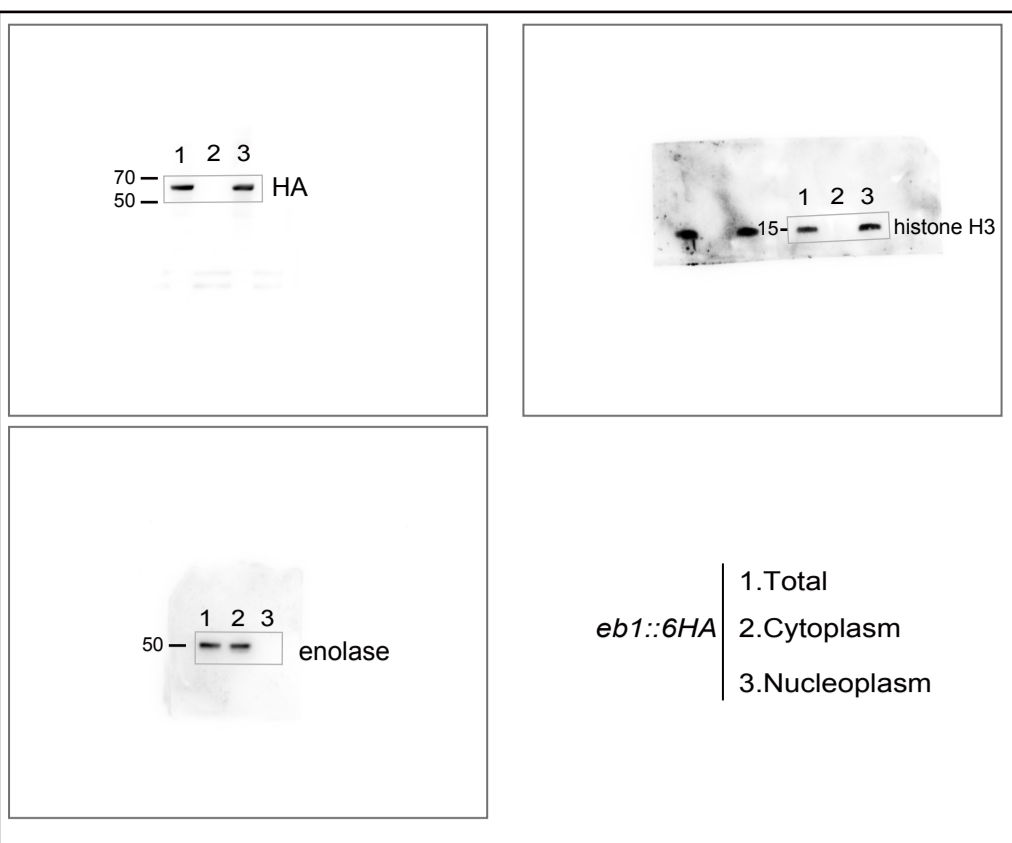

Fig. 1J

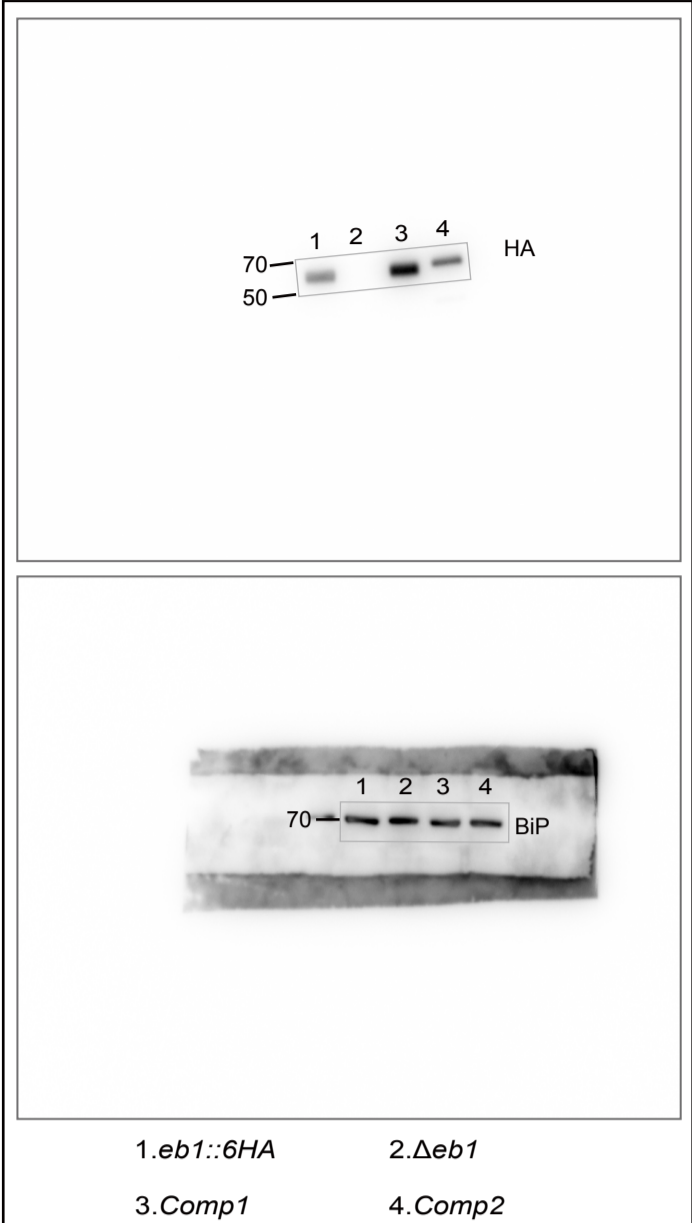

Fig. 5B

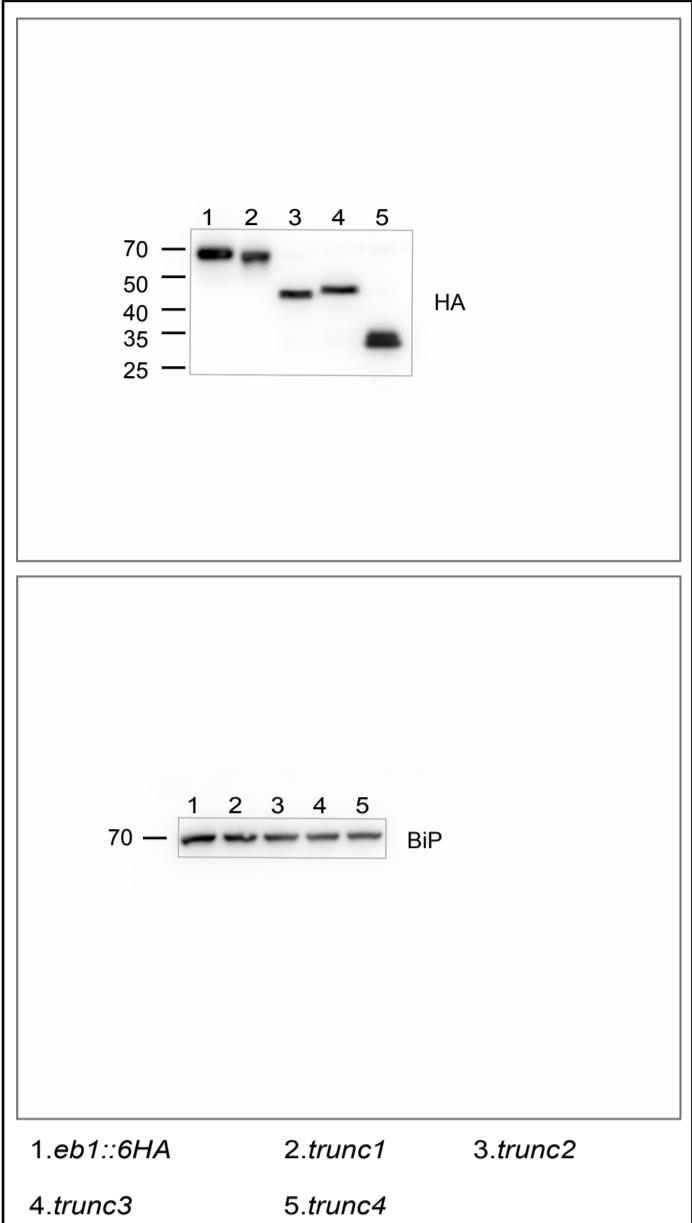

Fig. 8B

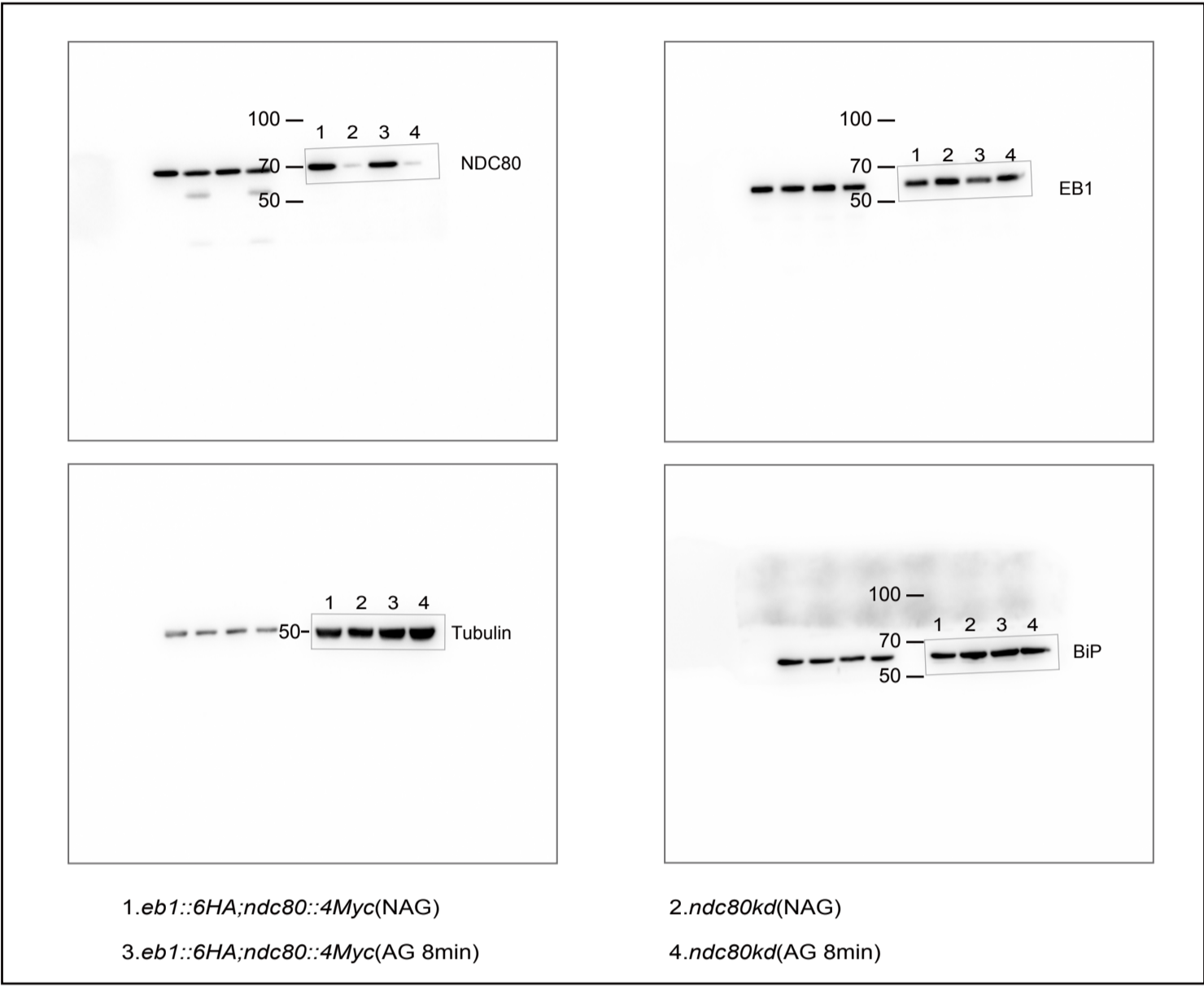

Fig. 9C

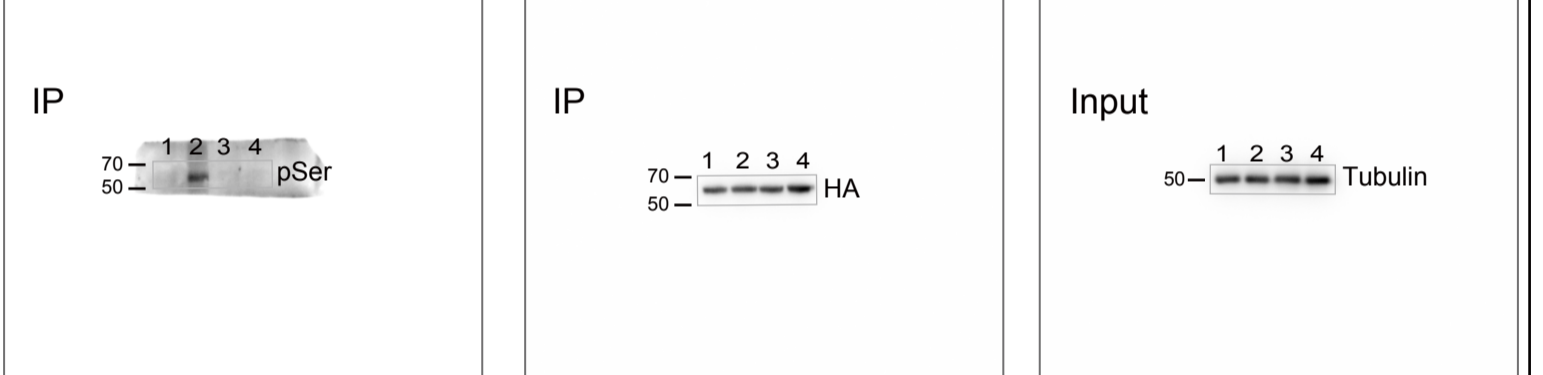

Fig. 9H

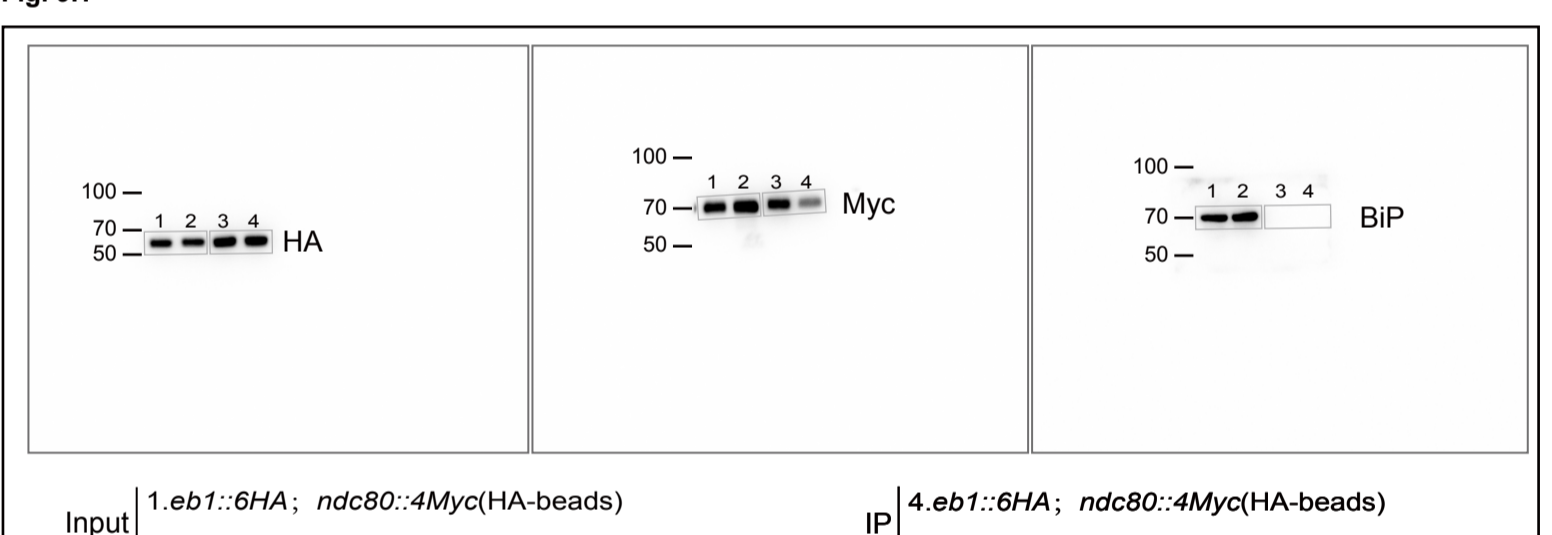

Fig. S8B

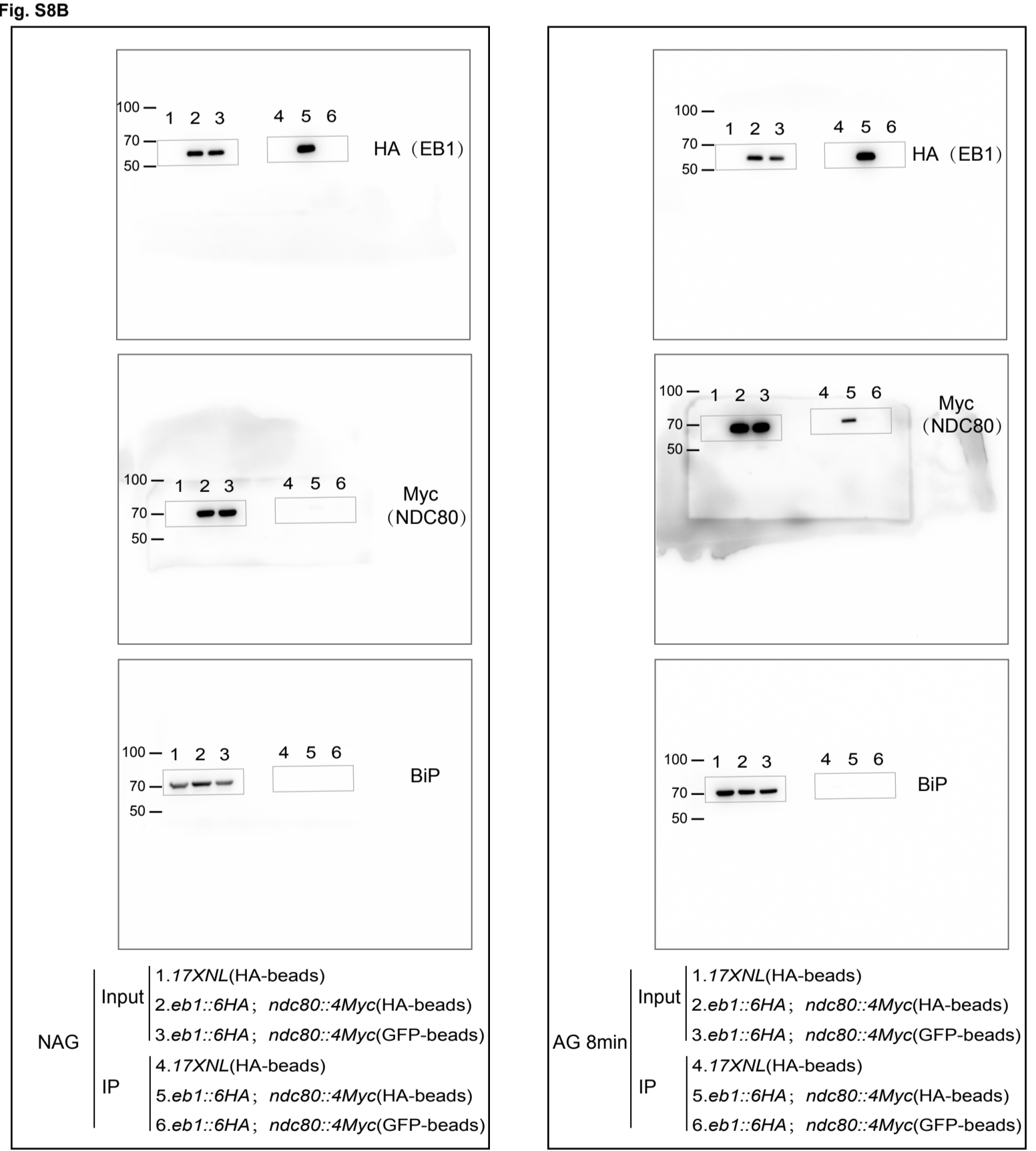

Fig. S8C

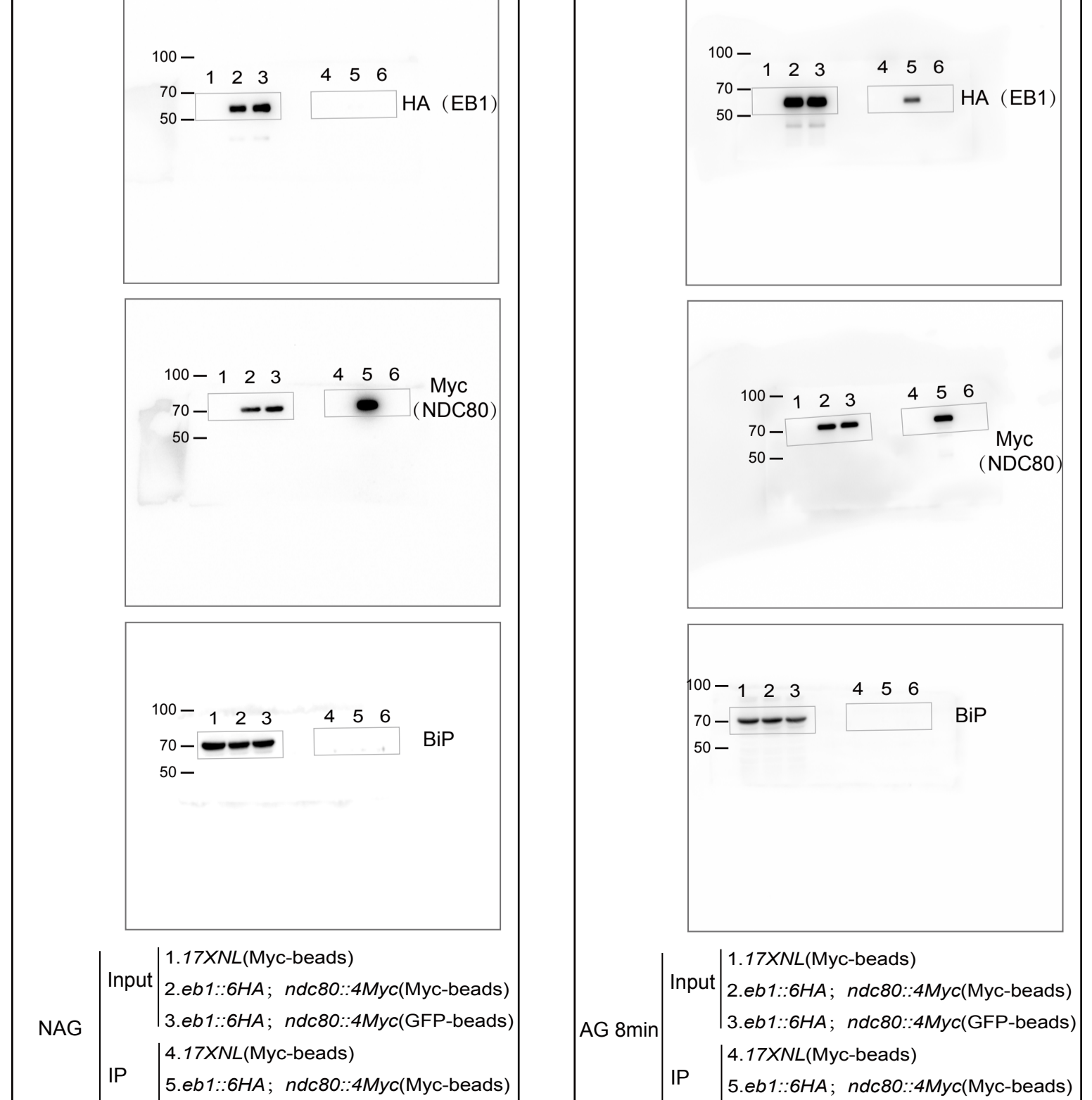

Supplement: Supplementary file 4 — Source data [file 41467_2023_38516_MOESM4_ESM.zip › WB full scan.pdf]
